# Exosomal telomerase transcripts reprogram the microRNA transcriptome profile of fibroblasts and partially contribute to CAF formation

**DOI:** 10.1038/s41598-022-20186-8

**Published:** 2022-09-30

**Authors:** Daniela Likonen, Maria Pinchasi, Einat Beery, Zinab Sarsor, Lorenzo Federico Signorini, Asia Gervits, Roded Sharan, Meir Lahav, Pia Raanani, Orit Uziel

**Affiliations:** 1 Present Address: The Felsenstein Medical Research Center, Petah-Tikva, Israel; 2 Present Address: School of Computer Science, Tel-Aviv University, Tel-Aviv, Israel; 3 Present Address: Sackler School of Medicine, Tel-Aviv University, Tel-Aviv, Israel; 4 Institute of Hematology, Davidoff Cancer Center, Rabin Medical Center, Petah-Tikva, Israel

**Keywords:** Cancer, Cell biology

## Abstract

It is now well accepted that cancer cells change their microenvironment from normal to tumor-supportive state to provide sustained tumor growth, metastasis and drug resistance. These processes are partially carried out by exosomes, nano-sized vesicles secreted from cells, shuttled from donor to recipient cells containing a cargo of nucleic acids, proteins and lipids. By transferring biologically active molecules, cancer-derived exosomes may transform microenvironmental cells to become tumor supportive. Telomerase activity is regarded as a hallmark of cancer. We have recently shown that the transcript of human telomerase reverse transcriptase (hTERT), is packaged in cancer cells derived- exosomes. Following the engulfment of the hTERT transcript into fibroblasts, it is translated into a fully active enzyme [after assembly with its RNA component (hTERC) subunit]. Telomerase activity in the recipient, otherwise telomerase negative cells, provides them with a survival advantage. Here we show that exosomal telomerase might play a role in modifying normal fibroblasts into cancer associated fibroblasts (CAFs) by upregulating $\mathrm{\alpha }$SMA and Vimentin, two CAF markers. We also show that telomerase activity changes the transcriptome of microRNA in these fibroblasts. By ectopically expressing microRNA 342, one of the top identified microRNAs, we show that it may mediate the proliferative phenotype that these cells acquire upon taking-up exosomal hTERT, providing them with a survival advantage.

## Introduction

The importance of the microenvironment for the development, perpetuation and spreading of the tumor mass has been extensively shown^1,2^. Secreted from most cell types, including cancer cells, exosomes are nanosized particles (30–150 in diameter) that mediate the cross talk between environmental and tumor cells. Exosomes carry a cargo of nucleic acids, proteins and lipids which mostly reflects the cellular content of their cells of origin. They travel in all body fluids and are taken up by cells in their vicinity. By transferring biologically active molecules to these recipient cells, cancer derived exosomes may transform microenvironmental cells to become tumor supportive cells, thus acting as intercellular communicators^3–6^. For example, they transform intact fibroblasts into cancer associated fibroblasts^7,8^. Additionally they are involved in processes such as metabolic reprogramming by which fast-growing cancer cells adapt to their increasing energy demands^9^ and evade the immune system^10^.

The activity of telomerase, a reverse transcriptase which elongates telomeres, is a hallmark of cancer cells. This is due to the enzyme's activity in the vast majority of malignant cells and its absence in most somatic cells^11^. Telomeres are repetitive sequences located at the ends of eukaryotic chromosomes, forming a 3D structure together with the shelterin protein complex, providing stability to the whole genome. They are getting shorter with each DNA replication accompanied to cell division and upon reaching a certain threshold they signal the cells to stop dividing and enter a senescence state. As such, they are considered a mitotic clock^12^. By preventing telomere shortening, the activity of telomerase provides cancer cells with unlimited replicative potential^13^. We have recently shown that exosomes derived from cancer cells contain the transcript of telomerase. When these exosomes were taken up by fibroblast, otherwise telomerase negative cells, the transcript was translated into a fully active enzyme, transforming these cells into telomerase positive cells, providing them with a survival advantage including the decrease of number of senescent cells and increase in cellular proliferation rate^14^. Apart from telomere synthesis, telomerase possess other "extracurricular" activities, which are telomere length independent, for example, prevention of apoptosis and a partial protection from DNA damage^15^. Another example is its involvement in microRNA upregulation which has been documented only once^16^. The goal of the current study was to explore whether some of the phenotypic changes carried out by exosome-derived telomerase activity in the fibroblasts were mediated by modulating these cells into cancer associated fibroblasts (CAFs) and by the upregulation of specific microRNAs in these cells.

## Materials and methods

### Cell lines, growth conditions and cell's transfection

The following cell lines were used in the study: Acute T cell leukemia (Jurkat, kindly provided by Dr. Galit Granot, FMRC), primary human foreskin fibroblast (pHFF, kindly provided by Dr. Selig from the Israel Institute of Technology); human foreskin fibroblast (hFF). The hFF cell line was immortalized by the ectopic expression of hTERT as previously described^17^ and kindly provided by Dr. Selig from the Israel Institute of Technology.

Jurkat cell line was cultured in RPMI-1640 with 20% fetal bovine serum (FBS), containing 100 units/ml L-Glutamine and 1% streptomycin (Biological Industries Beit Haemek, Israel). hFF and pHFF cells were cultured in Dulbecco's minimal essential medium (DMEM) with 20% FBS containing 100 units/ml L-Glutamine and 1% streptomycin (Biological Industries). The cells were incubated at 37 °C in 5% CO_2_. For exosome isolation, an exosome free media was prepared by ultracentrifugation of FBS at 100,000 g for 16 h at 4 °C (Optima™ XPN, Beckman coulter, USA).

For cell transfection, 2.5 $\times {10}^{5}$ pHFF cells/well were seeded in 6-well plate for 24 h and were subsequently transfected by using HiPerFect transfection reagent (Qiagen, Germany) according to the manufacturer’s instructions. Briefly, miRNA mimics was diluted (150 ng) in 100 µl of culture medium without serum for a final concentration of 5 nM. Then it was mixed with 12 µl of HiPerFect transfection reagent to allow the formation of transfection complexes in room temperature for 10 min. The resulting transfection solutions were then added to each well containing 2.3 ml of culture medium. Cells were grown at 37 °C for 72 h before another transfection was performed as above.

Cells were transfected with the following miScript miRNAs:

- hsa miR-342-3p miRNA (miR342 mimic)
- miRNA Negative Control with a scrambled sequence.
- miRNA Inhibitor
- miRNA Inhibitors Negative Control

### Isolation of exosomes

Exosomes were isolated by serial ultracentrifugation as previously described^18^. Briefly, a total of 5× ${10}^{7}$ Jurkat cells were grown in exosome depleted media for 3 days. Then, the cells debris was removed by 2 consecutive centrifugations at 1000 RCF and 2000 RCF each. Subsequently, the media was centrifuged at 10,000 RCF for 30 min at 4 °C and the supernatant collected and filtered through 0.22 µm filters (Merck Millipore, USA). The clarified medium was than centrifuged at 100,000 RCF at 4 °C for 70 min (Beckman Coulter, CA, USA). The crude exosome-containing pellets were resuspended in 1 mL and a second round of ultracentrifugation at 100,000 RCF at 4 °C for 120 min was carried out. The resulting exosome pellet was resuspended in 200 µL of PBS and was referred to as the first pellet. A second pellet was obtained after centrifuging the supernatant of the first pellet at 100,000 RCF at 4 °C for 120 min.

### Nanosized tracking analysis of Jurkat derived exosomes

Exosomes were quantified by using the Nanosight tracking analysis, according to the manufacturer's instructions (Malvern Panalytical, Cambridge UK) and following published method^19^. The analysis settings were optimized and kept constant between samples.

### BCA Protein quantification

Exosomes were suspended in RIPA buffer and centrifuged for protein extraction. Total protein was quantified using Pierce BCA Protein Assay Kit (Thermo Scientific, MA, USA) in accordance with the manufacturer's instructions.

### Exposure of fibroblasts to Jurkat derived exosomes

200μls of the resuspended exosomes were added to 3X10^5^ pHFF cells per well in a 6-well culture plate. 6 h later the fibroblasts were harvested and RNA was extracted for the measurement of hTERT expression by q-PCR. Previous studies conducted in our laboratory have suggested that exosomes affected the recipient cells in a dose and a time dependent manner. Therefore, we used these conditions in all further experiments.

### RNA isolation

Total RNA was extracted using the EZ-RNA II Isolation Kit reagent (Biological Industries Beit Haemek, Israel) according to manufacturer's instructions. Briefly, cells were lysed with guanidine thiocyanate detergent solution, followed by organic extraction and alcohol precipitation of the RNA. Quantification and the purity of total RNA were assessed by using the NanoDrop1000® spectrophotometer (Thermo Fisher Scientific, MA, USA).

RNA isolation from exosomes was performed by using Total Exosome RNA and Protein Isolation Kit (Invitrogen, CA, USA). Exosomes were lysed with denaturing solution and then precipitated by acid-phenol: chloroform solution followed by several washes and centrifugation steps. The supernatant was then placed on filter cartridge tubes for further purification using numerous washing solutions provided by the kit. RNA was eluted with PCR grade water.

### cDNA formation

Total RNA extracts from cells were reverse transcribed using the High-Capacity cDNA Reverse Transcription Kit (Applied Biosystem, CA, USA) according to the manufacturer's instructions. Briefly, 1000 ng of template RNA was added to 10μL reaction mixture containing dNTP's, random primers, RNase inhibitor, reaction buffer and reverse transcriptase. The incubations steps included 10 min at 25 $^\circ$C followed by 120 min at 37 $^\circ$C and the reaction was terminated by a heating step at 85 $^\circ$C for 5 min.

### Q-real time PCR reaction

Real-time-PCR was employed to estimate the levels of the hTERT mRNA in exosomes secreted from the growth media of Jurkat cells based on the Taqman methodology (Applied Biosystems, CA, USA) and for the validation of the results of the microRNAs analysis. The expression of the HPRT-1 (Hypoxanthine–guanine-phosphoribosyltransferase-1) gene was used as the endogenous control. PCR reactions were carried out using the Step One Plus detection system (Applied Biosystems, CA, USA). Taqman real time PCR reactions were performed with 50 ng of cDNA, specific primers and Taqman gene expression master mix (Applied Biosystems, CA, USA). The incubations steps included 2 min at 50 $^\circ$C followed by 10 min at 95 $^\circ$C and 40 cycles of 15 s at 95 $^\circ$C and 1 min at 60 $^\circ$C.

PCR reactions were prepared with the following custom made Taqman fluorochrome labelled primers (Applied Biosystems, CA, USA).

hTERT primers: Forward: 5′-CGTCCAGACTCCGC TTCATC-3′

Reverse: 5′-GAGACGCTCGGCCCTCTT-3′;

HPRT-1 primers: Forward: 5-′TTATGGACA GGACTGAACGTCTTG-3′

Reverse: 5′-TGTAATCCAGCAGGTCAGCAAA-3′

### Western blot

The expression of CAFs markers in exosomal exposed fibroblasts was assessed by Western immunoblot analysis. Proteins were extracted and quantified using the Bradford assay (Bio-Rad Laboratories, CA, USA). 25 μg of proteins extracted from treated and untreated cells were subjected to Poly-Acrylamide Gel Electrophoresis and transferred to a nitrocellulose membrane. Membrane was hybridized with antibodies against the different proteins: αSMA (1:500, Bio-Rad, California, USA); Vimentin (1:200, Santa Cruz Biotechnology, Texas, U.S.A.); and subsequently to fluorescent labeled secondary antibodies. Visualization was done by the Odyssey analysis software (Odyssey IR imaging system; LI-COR).

### ELISA assay

The expression of IL-6 by exosome exposed fibroblasts was evaluated by ELISA assay. Culture supernatants were collected centrifuged to pellet any detached cells and analyzed using a Human IL-6 Quantikine ELISA Kit (R&D Systems Inc., MN, USA) according to the manufacturer's instructions. Culture supernatants were diluted 150- 1000-fold for IL-6 measurements.

### miRNA profiling

microRNA expression levels were determined by using the Rosetta Genomics proprietary microarrays platform containing 2167 microRNA probes and various control probes. RNA was extracted and labeled; samples were processed according to standard protocols. Data was stored in the Microarray Database. Statistical probability of significance (adjusted p values, set at p < 0.05) and a fold change of > 2 were used to select the miRNAs that were significantly differentially expressed between our samples. The miRBase website^12^ was used to retrieve the mature sequences for each miR.

### Validation of the results of the microRNA analysis

q-PCR was performed to validate and quantify the expression of the results of the microRNA analysis (miR-342-3p, miR-125b-1, miR-128-3p and miR-92a-3p). Prior to RNA isolation as described above, pHFF cells were exposed to Jurkat cells derived exosomes for 6 h and the levels of microRNA were determined by Q-PCR. pHFF cells without exosome exposure served as controls. Specific commercial primers sequences were used for the validation of each miRNAs (ABI, Foster City, CA, USA).

### miRNA cDNA formation

For miRNA-specific cDNA formation we used 20 ng total RNA from the RNA extraction samples. Taqman® small RNA Assay was used for miRNA reverse transcription reaction. 1–10 ng of total RNA sample were used per 15 µl RT reaction. Taqman® MicroRNA probe was used as the Reverse Transcription primers. Each reaction included 7 µl master mix, 3 µl 5 X RT primers (miRNA specific primers) and 5 µl of RNA sample (1–10 ng).

### miRNA Q-real time PCR reaction

Taqman® Small RNA Assays kit was used for Real-time quantitative PCR according to the provided manual (ABI, CA, USA), run and analyzed by the Step- One Q-PCR device (ABI).

miRNA specific primers were chosen for the following miRNAs: miR-342-3p, miR-125b-1, miR-128-3p and miR-92a-3p. U3 was selected as a housekeeping gene to normalize the miRNA levels.

The incubation steps included: 10 min at 95 °C, followed by 40 cycles of 15 s at 95 $^\circ$C and 60 s at 60 °C.

### Proliferation assay

Cellular proliferation of pHFF cells was assessed by the Sulphorodamine B (SRB) assay. Post transfection, ${10}^{4}$ cells/ml were cultured in 24-well plates for 72 h. Cells were then fixated to the plate with 10% Tetrochloric Acid and incubated at 4 $^\circ$C for 1 h, then washed with dH_2_O. Subsequently, 1 ml of SRB coloring agent (Sigma Aldrich Israel Ltd., Rehovot, Israel) was added to the wells which were incubated at room temperature for 30 min and then washed with 1% acetic acid. After adding 10 nM TRIS^.^HCl buffer absorbance was measured at 515 nm by an ELISA microplate reader (Biotek Instruments Inc., VT, USA). Experiments were performed in triplicates.

### Cell cycle analysis

The cell cycle status was analyzed by flow cytometry. Cells were processed by standard methods using propidium iodide staining of the DNA. Briefly, after cells' transfection upon reaching 60% confluence, the cells were trypsinized, resuspended in 5 ml cold PBS and centrifuged at 2500 rpm for 5 min at 4 °C. Cells were subsequently pelleted and fixed by adding 4.5 ml 70% cold ethanol dropwise to 0.5 cold PBS, with gentle mixing. The cells were then incubated for > 2 h at -20 °C. Cells were subsequently washed by PBS and centrifuged at 2500 rpm for 5 min 4 °C. Staining with propidium iodide (P4170, Sigma Aldrich) was conducted prior to flow cytometry analysis. Cells were pelleted and resuspended in 0.5 ml PBS containing 0.025 ml PI (50 μg/ml in PBS and 5 μl of 100 mg/ml DNase-free RNase (Sigma, St Louis, MO). Cells were then incubated at 37 °C for 15 min. Samples were analyzed on a Beckman-Coulter Epics XL-MCL apparatus. The parameters were adjusted for the measurement of single cells using the forward and side scatter plots.

### Prediction of miRNA targets

To determine potential mRNA targets for specific miRNAs we have used several publicly available target prediction algorithms based on sequence complementarity between the miRNA and its potential mRNA target 3′ untranslated region (3′UTR).

TargetScan^20^ was used to determine potential miR-342 targets involved in cell proliferation and cell cycle. In addition to TargetScan, a combination of bioinformatics tools for miRNA target prediction, including miRBase^21^, miRanda^22^, DIANAmt^23^, miRDB^24^, miRWalk^25^ and PITA^26^ were used.

### Functional annotation and gene set enrichment analysis

Several bioinformatic tools have been used for the biological interpretation and the analysis of pathways associated with defined lists of genes. These included DIANA miRPath and DAVID. mirPath is a web-based computational tool developed to identify molecular pathways potentially altered by the expression of single or multiple microRNAs. The software performs an enrichment analysis of multiple microRNA target genes comparing each set of microRNA targets to all known pathways. Pathways with p-value < 0.05 providing statistically significant values are selected^27^. mirPath was used to gain insight into global molecular networks and canonical pathways specific to miR-342.

The Database for Annotation, Visualization and Integrated Discovery (DAVID) v6.7 bioinformatic algorithm^28^ was used for the functional annotation analysis of Gene Ontology (GO) terms associated with a given gene list of confirmed mRNA targets within each miRNA cluster specific for miR-342. We analyzed the list of the most significantly regulated genes using the ClueGO Cytoscape plugin^29^. GO functional categories with p-values under 0.05 were considered statistically significant. To study the putative interactions between the four selected miRs and telomerase, we identified proteins that are upstream to these miRs. These proteins were subjected to the Advanced Network Analysis Tool (ANAT) software, a tool for constructing and analyzing functional protein networks. ANAT provides access to up-to-date large-scale physical association of protein data, advanced algorithms for network reconstruction and several tools for exploring and evaluating the obtained network models. We used the network-based analysis inferring an anchored network (telomerase) that connects a given set of proteins (proteins that regulate the four selected miRs expression) to designated anchor set of proteins^30^.

### Statistical analysis

Quantitative data were expressed as means ± SD. Statistical significance was determined by the Student’s t-test. P-value < 0.05 was considered as statistically significant.

Statistical analysis for the data presented in Figs. 2, 4, 5 and 7a was conducted with SAS version 9.4, using ANOVA with post- hoc comparisons with Tukey adjustment for multiple comparisons.

## Results

### Jurkat cells derived exosomes are taken up by fibroblasts

**Figure 1:**
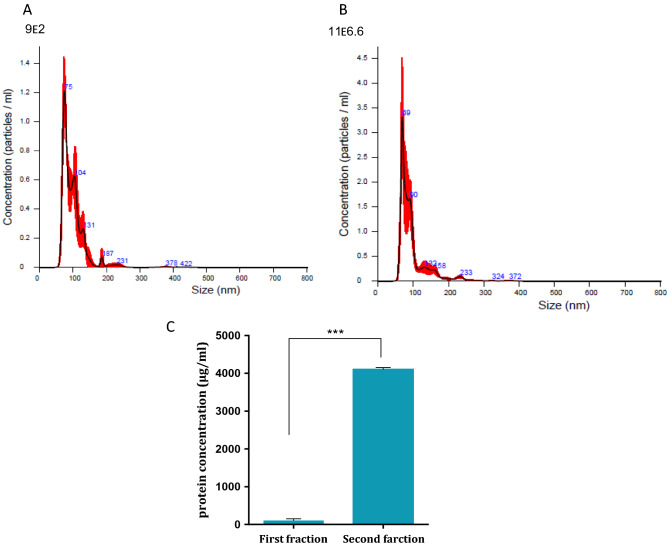
Quantification of Jurkat cells exosomes purification. Jurkat cells exosomes were purified from two fractions. The two fractions were quantified using the Nano- Sight tracking analysis (NTA). Represented images of the quantification of the first fraction (using dilution factor 1:200) (**A**) and the quantification of the second fraction (using dilution factor 1:6600) (**B**) are shown. (**C**) Quantification of total protein concentration of the two fractions of purified Jurkat cells exosomes. The graphs values represent mean concentration ± S.E of three independent experiments. *Indicates PV < 0.001. D. Examples of single exosomes images captured by EM.

**Figure 2:**
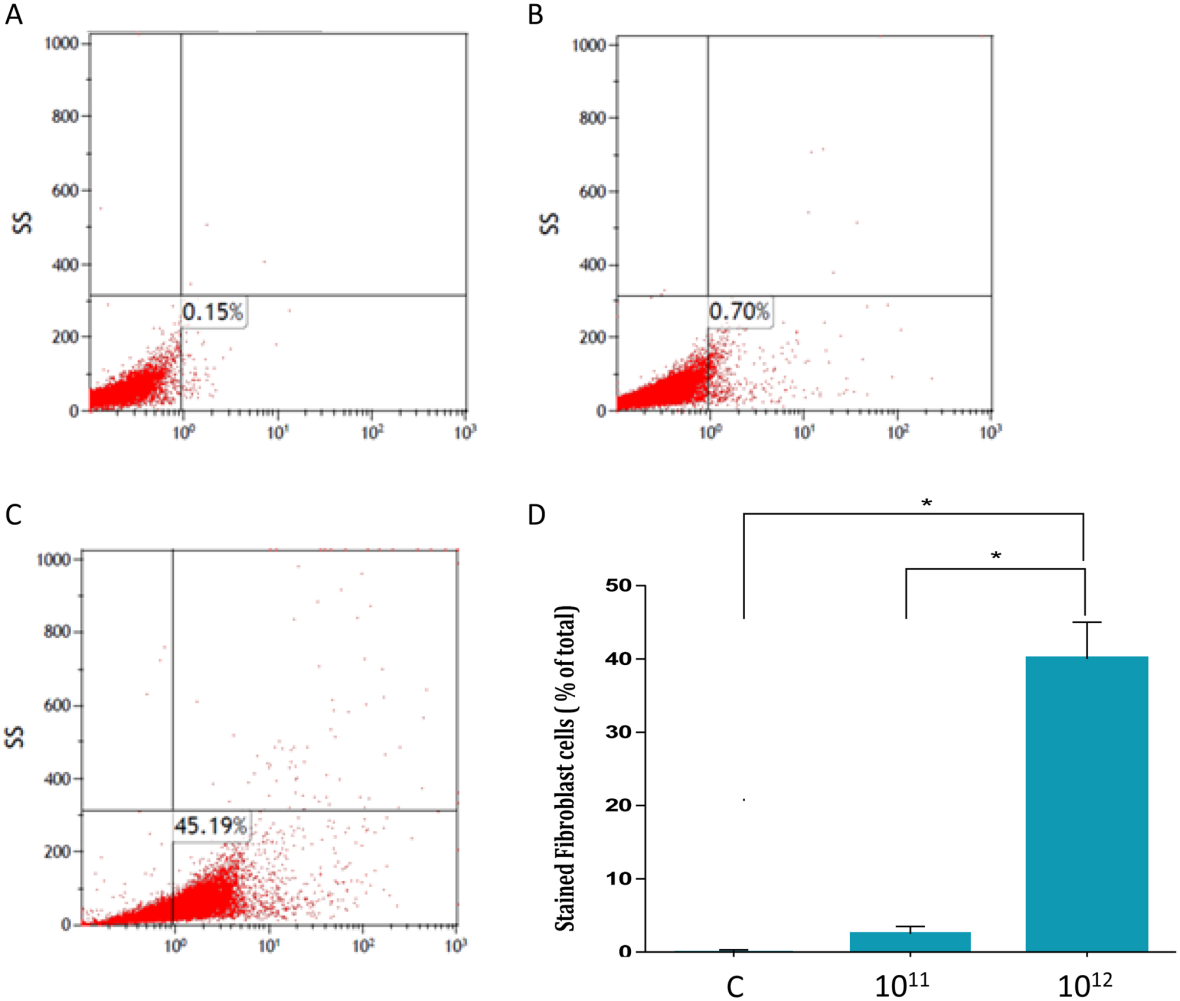
Jurkat cells derived exosomes uptake by fibroblasts. Jurkat cells derived exosomes were labeled using FM 1–43 membrane dye. Fibroblasts were treated with several exosome concentrations for 24 h. Exosomal uptake was determined using FACS analysis. X axis: percent of fibroblasts labeled with FM 1–43. (**A**) Control fibroblasts. (**B**) Fibroblasts treated with 10^11^ exosomes. (**C**) Fibroblasts treated with 10^12^ exosomes. Represented images are shown. (**D**) Quantification of exosomal uptake. The graphs values represent mean (%) ± S.E of three independent experiments. * indicates PV < 0.05.

To harvest Jurkat cells derived exosomes we grew the cells in exosomes free medium for 72 h. Exosomes were isolated using differential centrifugation. Two fractions were analyzed: the pellet of exosomes at the end of the ultracentrifugation process and the supernatant containing exosomes of the pellet. As shown in Fig. 1, nanosized tracking analysis (NTA) revealed that the second fraction contained more exosomes which fall into a range of 30- 150 nm, the expected size of exosomes. An example of the exosomal fraction is shown in Fig. 1B, in which the highest exosomal amount picked at 69 nm. (Protein analysis confirmed the NTA results (Fig. 1C). Therefore, throughout the study we have used the second exosomal fragment. These results verified that Jurkat cells derived exosomes could be isolated. Subsequently, we exposed fibroblasts to the isolated exosomes. Jurkat cells exosomes were labelled with FM 1–43 membrane fluorescent probe. The labeled exosomes were added to fibroblasts in a dose dependent manner while control cells were treated with PBS only. FACS analysis was performed 24 h afterwards. As demonstrated in Fig. 2, the FM 1–43 dye labeled fibroblasts population was increased from 0.15% to 0.7% when 10^11^ exosomes were applied and to 45.19% when 10^12^ exosomes were used (Fig. 2C). Previous studies conducted in our laboratory showed that the optimal uptake time of these exosomes was 24 h (not shown).

### Jurkat cells derived exosomal hTERT modify CAF related markers to a certain extent

**Figure 3:**
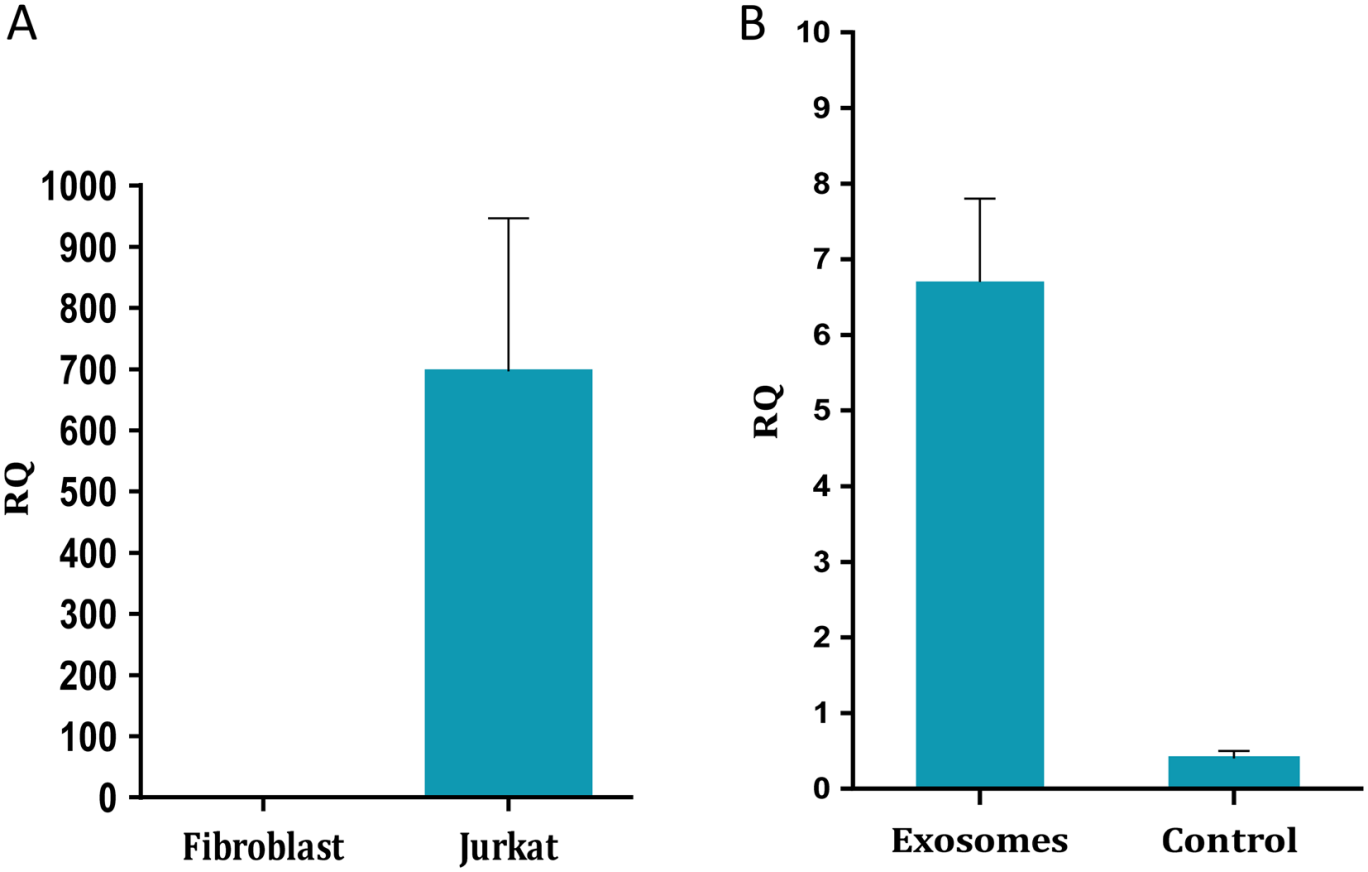
hTERT expression in Jurkat cells exosomes and in subsequent fibroblasts. A. Exosomal RNA was isolated from three different exosome isolations. hTERT and HPRT-1 as a control gene were amplified by Real-Time PCR. The Y axis represents the normalized hTERT expression level in Jurkat cells exosomes compared to control fibroblasts exosomes. For calculation needs, a threshold of Ct 50 was used as the "undetectable" hTERT expression in fibroblasts. The RQ values were calculated based on the 2$\Delta \Delta$^ct^ and were multiplied by 100. The bars represent average hTERT expression of each sample ± S.E of three independent experiments. B. 3 × 10^5^ pHFF cells/ml cells were subjected to Jurkat-derived exosomes for 6 h. The cells were harvested and the expression of the hTERT mRNA was carried out by quantitative real time PCR. The control cells are those that were exposed to PBS only. Each column represents mean ± S.E of three independent experiments. Exosomes: cells exposed to Jurkat derived exosomes, control- cells exposed to PBS only.

In a previous study we showed that exposure of fibroblasts to exosomes derived from Jurkat cells induced about 3.5-fold expression of hTERT in the recipient's previously telomerase negative cells^14^. These exosomes were previously isolated using a commercial kit. We repeated this experiment by using exosomes that were isolated by serial centrifugation and as shown in Fig. 3A the cargo of these exosomes contained the transcript of the hTERT gene. An increase of about 13-fold in the expression of the transcript of telomerase was detected 6 h post exosomal exposure (Fig. 3B). As shown in our previous study, the hTERT transcript was translated into a fully active enzyme in the recipient's fibroblasts and after assembly with its RNA component hTERTC to form an active enzyme, the phenotype of these cells was transferred from telomerase negative into telomerase positive cells.

**Figure 4:**
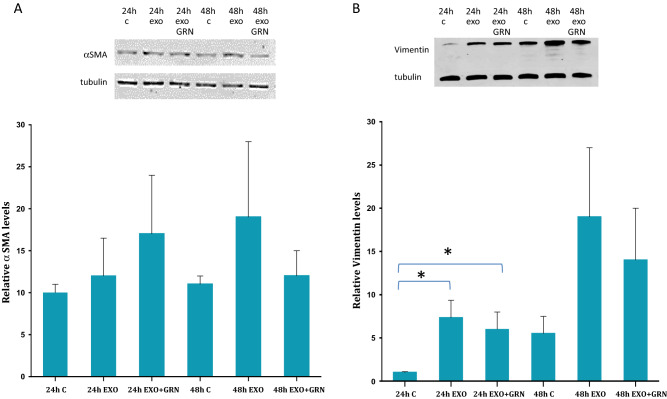
CAFs markers levels in exosome exposed fibroblasts. Fibroblasts were exposed to Jurkat cells derived exosomes (Exo), to telomerase inhibitor (GRN) or to Jurkat-derived exosomes and telomerase inhibitor (Exo + GRN) for 24 and 48 h. Cells were harvested, lysed and proteins were extracted. 25 μg of protein lysate was subjected to Western blot analysis to determine possible alterations in the expression levels of the CAFs markers: (**A**) αSMA and (**B**) Vimentin. Upper panel: example of one representative Western blot. Lower panel: Quantification of the cellular levels of the analyzed proteins. Each column represents mean (%) ± S.E of three independent experiments. *Indicates PV < 0.05.

Cancer cells' exosomes induce the generation of cancer associated fibroblasts (CAFs) in various experimental systems^31,32^. To understand whether some of CAFs markers are upregulated in response to telomerase activation, we exposed the fibroblasts to Jurkat derived exosomes with or without the telomerase inhibitor GRN163L. As demonstrated in Fig. 4A and suppl. Figure 1, the exposed fibroblasts exhibited a slight insignificant increase of 133% in αSMA levels after 24 h. This elevation was not telomerase dependent as the inhibition of telomerase in the recipient fibroblasts did not reduce the levels of αSMA. However, 48 h after exosomal exposure, the increase in αSMA was decreased when telomerase was inhibited, pointing to a non-significant statistical trend of the levels of αSMA which may be interpreted as telomerase activity dependent.

A similar result was obtained when we analyzed the levels of vimentin. Twenty-four hours post exosomal exposure the levels of vimentin increased to 540% but were unchanged in response to the inhibition of telomerase. Forty-eight hours post exosomal exposure the levels of vimentin increased by 344%. These levels decreased to 251% upon telomerase inhibition. Although only part of these changes reached statistical significance, a non- significant trend may be described, which might be interpreted as a partial non-significant dependency of the levels of vimentin in telomerase (Fig. 4B, Suppl. Figure 1). To gain more insight into possible connection of these CAF markers and telomerase, we employed the Advanced Network Annotation Tool (ANAT) software^24^. Based on databases of protein–protein interactions, ANAT connects a group of selected proteins (in our case- the identified CAF markers) to an "anchor" protein (human telomerase). The associations that are formed by ANAT are all statistically significant (compared to random input connections). This analysis identified a single pathway connecting telomerase and the CAF markers, Vimentin and αSMA, as follows: TERT- AKT-1- Vimentin- GABARAP-1- αSMA.

These associations actually describe a direct biological link between telomerase, Vimentin and αSMA.

### Fibroblasts exposed to Jurkat cells derived exosomes secrete IL-6

**Figure 5:**
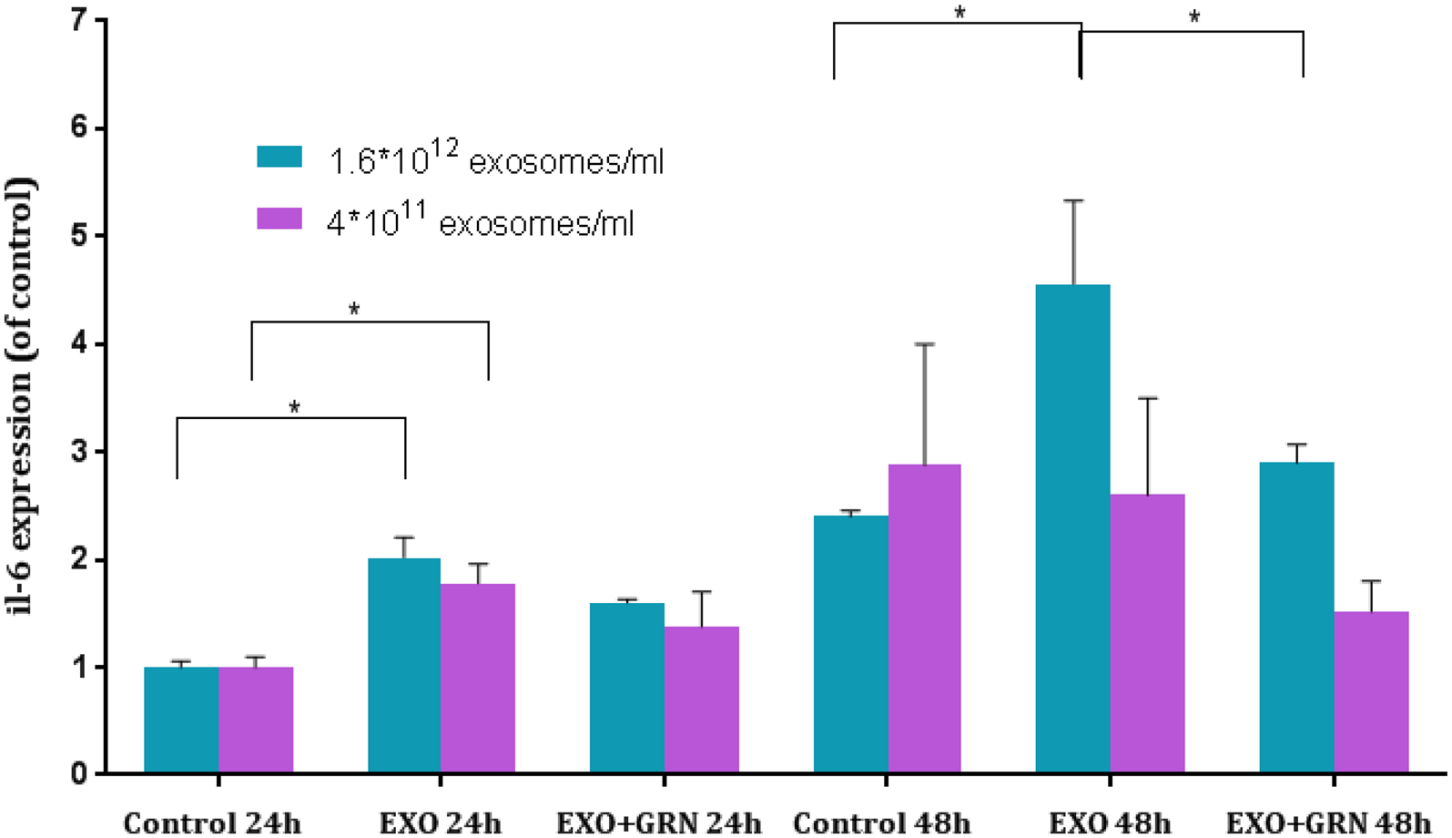
IL-6 secretion from exosome treated fibroblasts. Fibroblasts were exposed to Jurkat cells derived exosomes (Exo) in two different concentrations, to telomerase inhibitor (GRN) or to Jurkat-derived exosomes and telomerase inhibitor (Exo + GRN) for 24 and 48 h. Media was collected and IL-6 protein levels in the supernatants were measured by ELISA. Each column represents mean (%) ± S.E of at least two independent experiments. *Indicates PV < 0.05.

We next studied the effect of newly expressed telomerase in fibroblasts on inflammation by assessing the expression of IL-6, as a key inflammatory cytokine. Two different concentrations of purified Jurkat cells derived exosomes were added to cells and equal volume of PBS was added to the control plates. The media was collected after 24, 48 and 72 h and subjected to human IL-6 immunoassay. As demonstrated in Fig. 5, when fibroblasts were treated with the lower exosome concentration, IL-6 expression was 1.76 fold (SEM = 0.12, p = 0.01) higher 24 h post exosomal exposure compared to unexposed fibroblasts. The effect was partially due to newly expressed telomerase since treated cells with telomerase inhibition exhibited a milder increase in IL-6 expression [1.37 fold (SEM = 0.3, p = 0.14)- higher then untreated fibroblasts]. The effect was abolished 48 h after exosomal exposure. When the exosome concentration was higher (fourfold) the expression of IL-6 increased by twofold (SEM = 0.21, p = 0.0017) after 24 h compared to untreated fibroblasts. By 48 h, the effect was slightly diminished and IL-6 expression was 1.88-fold (SEM = 0.6, p = 0.036) higher in exosome treated fibroblasts compared to untreated fibroblasts. In each time point the effect was telomerase mediated to some degree since it was milder in treated fibroblasts with telomerase inhibition compared to exosomal exposed cells, although did not reach statistical significance in all cases (Fig. 5). After 72 h of exposure, the lower exosomal concentration did not affect the levels of IL-6 by the recipient cells, but the higher exosomal concentration resulted in a similar level to that of the 48 h effect (data not shown).

### Jurkat cells derived exosomes modulate the microRNA profile of pHFF cells

In light of the study by Lassmann et al., describing the role of telomerase in microRNA upregulation^16^, we sought to explore whether exosomal telomerase activity provides the recipient cells with a survival advantage, at least in part through the upregulation of specific microRNAs.

To test this hypothesis we exposed pHFF cells to Jurkat cells derived exosomes in the presence or absence of telomerase inhibitor, GRN163L. We and others have previously shown ~ 80–90% inhibition of the activity of the enzyme in response to that inhibitor^33,34^. We studied the profile of microRNA under six conditions:

- Jurkat cells cultured in exosome depleted medium.
- pHFFs cells, naturally devoid of telomerase activity and hTERT expression.
- pHFFs exposed to exosomes derived from Jurkat cells.
- pHFFs exposed to exosomes derived from Jurkat cells with inhibited telomerase activity. For this purpose, pHFF cells were cultured in the presence of GRN163L before Jurkat cells derived exosomal exposure.
- human foreskin fibroblasts (hFF) immortalized by the ectopic expression of hTERT- used as a positive control.
- Exosomes isolated from Jurkat cells grown in exosome depleted media.

Next, we compared the microRNA profile of the cells under the above experimental setting.

#### The effect of Jurkat derived exosomes on microRNA expression of fibroblasts cells

When comparing fibroblasts with or without exosomes derived from Jurkat cells, the following microRNAs were significantly upregulated: hsa-miR-92a-3p, hsa-miR-128-3p, hsa-miR-342-3p, hsa-miR-125b-1-3p, (Supp. Figure 2A, Table 1).

**Table 1:** Relative expression of top scored microRNAs in various setting.

microRNA/setting	Fold change	P Value	microRNA/setting	Fold change	P Value
**Fibroblasts exposed to Jurkat- derived-exosomes versus fibroblasts**			**Fibroblasts exposed to Jurkat- derived-exosomes and telomerase inhibitor versus intact fibroblasts**		
hsa-miR-92a-3p	3.9	0.003	hsa-miR-181a-5p	6.7	0.021
hsa-miR-128-3p	2.8	0.006	hsa-miR-378a-3p	11.5	1E-05
hsa-miR-342-3p	2.25	0.037	hsa-miR-4730	12.8	0.011
hsa-miR-125b-1-3p	2.2	0.045	hsa-miR-6132	6.9	0.046
**Fibroblasts exposed to Jurkat-derived exosomes and telomerase inhibitor versus fibroblasts exposed to Jurkat-derived exosomes**			hsa-let-7e-5p	0.2	0.045
hsa-miR-491-5p	3.3	0.01	MID-20094	0.1	0.018
hsa-miR-665	2.4	0.005	hsa-miR-664b-3p	0.1	0.024
hsa-miR-4730	19.9	0.002	**Fibroblasts with ectopically expressed telomerase versus intact fibroblasts**		
hsa-miR-371b-5p	4.8	0.013	hsa-miR-146a-5p	23.8	0.0015
hsa-miR-30a-5p	0.5	0.044	hsa-miR-490-5p	2.4	0.0006
hsa-miR-4284	0.5	0.008	hsa-miR-31-5p	0.4	0.0056
			MID-20524	0.5	0.011
			hsa-miR-409-3p	0.4	0.0024

**Figure 6:**
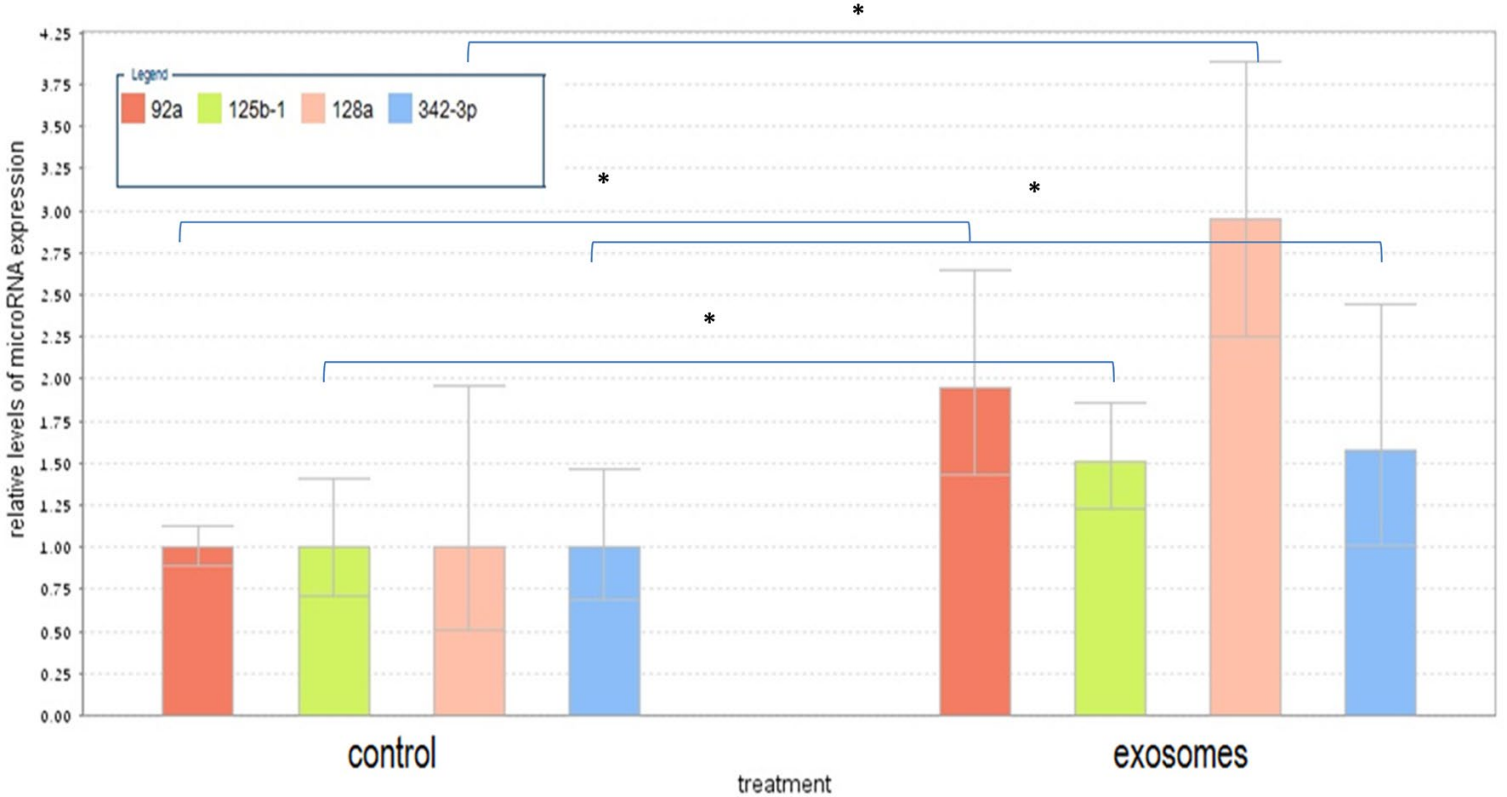
Q-Real-time PCR validation of the microarray data. Expression levels of miRNAs were measured by quantitative RT-PCR (Q-RT-PCR) in a subset of 4 samples. Cycle threshold values for each miRNA were normalized versus those of the housekeeping small RNA U3 gene. Q-RT-PCR showed that all four selected miRNAs display higher expression in the relevant samples after Jurkat cells derived exosomal exposure. Quantitative RT-PCR data are represented as the mean ± SD; n = 3. The statistical significance was *p < 0.05 as measured using the Student-t test.

We validated the expression of each of these microRNAs separately by Q-RT-PCR. As shown in Fig. 6, the expression of all four microRNAs was elevated upon exposure of fibroblasts to Jurkat cells derived exosomes.

#### The effect of telomerase inhibition on the expression of microRNAs in fibroblasts exposed to Jurkat cells derived exosomes

To understand the putative contribution of exosomal hTERT to microRNA expression in the recipient fibroblasts, we compared the expression of microRNAs in fibroblasts exposed to exosomes with or without telomerase activity. As shown in Supp. Figure 2B, the expression of four microRNAs was significantly upregulated and the expression of two microRNAs was downregulated as a result of the inhibition of telomerase activity in the recipient cells. These include: hsa-miR-491-5p hsa-miR-665 hsa-miR-4730 hsa-miR-371b-5p that were upregulated and hsa-miR-30a-5p and hsa-miR-4284 whose expression decreased in response to Jurkat cells derived exosomes (Table 1).

Since miR- 342 was not upregulated as in cells that were exposed to Jurkat- derived exosomes upon telomerase inhibition and actually returned to base line levels and due to its known involvement in oncogenesis we selected it for the next parts of the study.

#### The effect of telomerase inhibition on the expression of microRNAs in fibroblasts

To gain more insight into microRNA expression resulting from exosomal telomerase we compared fibroblasts that were exposed to exosomes and inhibition of telomerase versus naïve fibroblasts. The expression of seven microRNAs was significantly changed when cells were put under these conditions. The following microRNAs were upregulated: hsa-miR-181a-5p; hsa-miR-378a-3p, hsa-miR-4730 and hsa-miR-6132. Three microRNAs were downregulated in this setting: hsa-let-7e-5p, MID-20094 and hsa-miR-664b-3p (Supp. Figure 2C, Table 1).

#### The effect of ectopic expression of telomerase on the microRNA profile of fibroblasts cells

We were able to compare the microRNA profile of fibroblasts that were transfected with a plasmid expressing the catalytic subunit of telomerase, hTERT, expecting that this comparison will serve as a positive control. Supp. Figure 2D shows the result of this analysis, pointing to other microRNAs that changed their expression in response to the overexpression of hTERT. These include two microRNAs whose expression was increased: hsa-miR-146a-5p and hsa-miR-490-5p. Three other microRNAs that decreased their expression are: hsa-miR-31-5p, MID-20524, hsa-miR-409-3p (Table 1).

#### Profile of MicroRNAs that were packaged from Jurkat cells into their exosomes

We compared the cellular microRNA content of Jurkat cells to their cognate exosome microRNAs (Supp. Figure 2E). One hundred and twenty-two microRNAs were differentially expressed in the exosomes compared to the cells, 33 of these microRNAs levels were higher in the exosomes than in the cells, implying a selective encapsulation of these miRs by exosomes. One of these higher expressed miRs was miR-451a which is implicated in several malignancies and proliferative processes^35^. This active packaging of microRNA molecules into exosomes may suggest the activity of a sorting mechanism which has functional implications.

### miR342 contributes to the proliferative rate and increases the S phase of the cell cycle of fibroblasts

**Figure 7:**
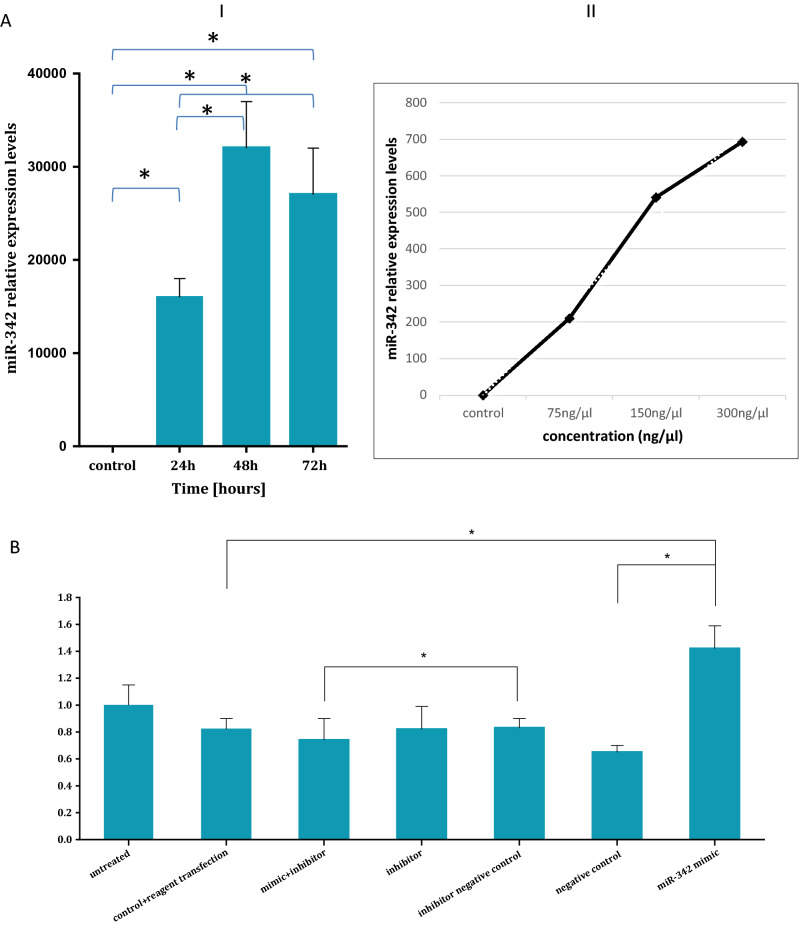

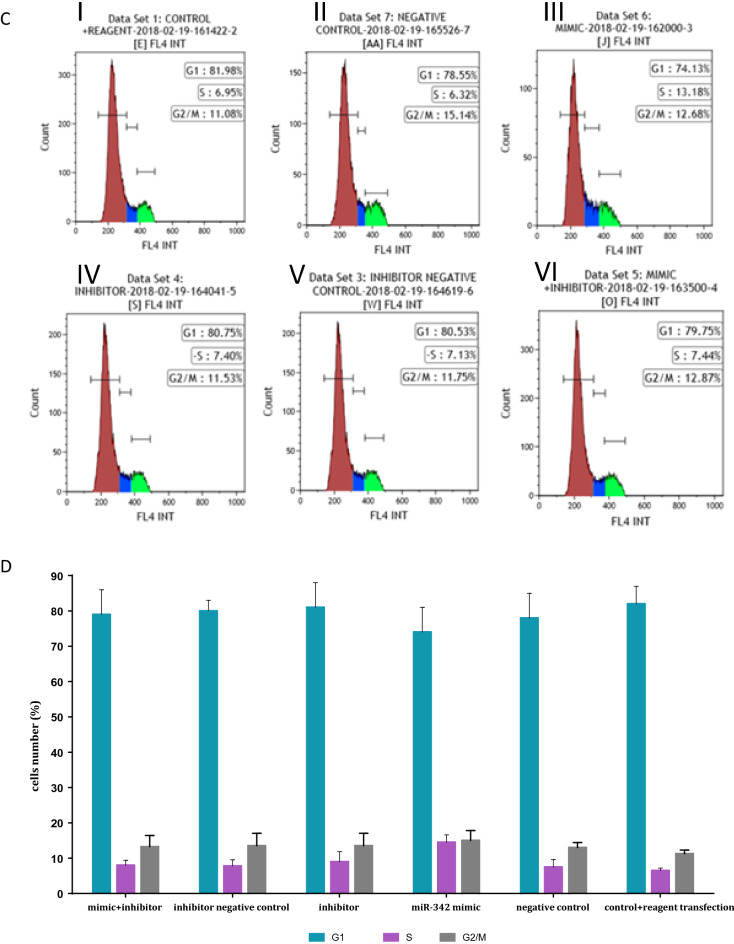
miR342 provides fibroblasts cells with a survival advantage. (**A**) Transfection of pHFF cells with miR342. Left panel (I): Q-RT-PCR analysis of the relative expression levels of miR-342 after its mimic transfection. The standard Taqman MicroRNA Assays was followed to examine performance of RNU6B (snRNA) endogenous control candidates served as an internal reference. The data summarizes three experiments and expressed as mean ± SD for three experiments. (II) Right Panel: Q- RT-PCR analysis of the relative expression levels of miR342 mimic in the pHFF cells after treatment with various concentrations of that miR-342 mimic (75 ng/μl, 150 ng/μl, 300 ng/μl). * indicates statistical significance. (**B**). MiR-342 overexpression increases cellular proliferation. 96 h after 150 ng/$\upmu$ LmiR mimic 342 and its various controls transfection, we observed a statistically significant increase in the growth of miR-342 overexpressing cells in comparison to cells treated by negative control, untreated fibroblast, or fibroblast + reagent transfection (p < 0.05) By using the SRC assay. The data summarizes three independent experiments and expressed as mean ± SD. (**C**) The cell cycle status of pHFF cells in response to the ectopic expression of miR342. The DNA content of the cells was measured by flow cytometry. Cells were processed by standard methods using propidium iodide staining of DNA. (I) pHFF cell-cycle profile of cells transfected with transfection reagent. (II) pHFF cell-cycle profile of cells transfected with negative control. (III) pHFF cell-cycle profile of cells transfected with miR-342 mimic (IV) pHFF cell-cycle profile of cells transfected with miR-342 inhibitor (V) pHFF cell-cycle profile of cells transfected with inhibitor negative control (VI) pHFF cell-cycle profile of cells transfected with miR-342 mimic + miR342 inhibitor. (**D**) Quantification of 7C. Graph displaying differences in cell cycle phases, as determined by flow cytometry analysis, between normal and miR-342 expressing pHFF cells. The data summarizes three independent experiments and expressed as mean ± SD.

Taken together, the results of the microRNA expression analysis under the various conditions point to miR342 as a potential contributor to the survival advantage of exosomally exposed fibroblasts. To study its putative role in cell proliferation we overexpressed its open reading frame in fibroblasts (miR mimic 342, Fig. 7AI and AII) and assessed its expression in a time and dose dependent manner. As a negative control to miR mimic342 we used a plasmid containing a scrambled oligonucleotide. In addition, we transfected the fibroblasts with an inhibitor of that miR and its negative control. Fibroblasts with no plasmid (only transfection reagent) were used as another negative control. Thereafter, we measured the proliferation rate of the resulting fibroblasts by using the SRB assay. A difference of approximately 1.5-fold of proliferation of pHFF cells containing ectopic miR-342 compared to that of the negative control was identified. The ectopic expression of miR-342 increased fibroblasts proliferation compared to cells in which the scrambled oligonucleotide was expressed (Fig. 7B).

To understand whether the increase in proliferation stems from change in the cell cycle of the exposed cells, we used the same experimental system and assessed the cell cycle status by flow cytometry. As shown in Fig. 7C,D, miR342 over expression resulted in G1/S transition in the exposed cells. The S phase of the cell cycle of cells exposed to miR342 was increased by two- fold compared to the control untreated cells (Fig. 7C). Whereas ~ 13% of the cells transfected with miR342 were in the S phase of the cell cycle, in all other controls (including the negative control and the inhibitor related controls) about 6.3–7.4% of the cells were in the S phase (Fig. 7D). All in all, these results suggest that miR-342 can increase the proliferation rate of fibroblast, thus providing them with a survival advantage.

One drawback of the current study is related to the ability to discriminate between telomere dependent and independent telomerase activities. Proliferation rate for example, can serve as the result of both activity types. This could have been done by following telomere dynamics in these cells after sufficient time of exosomal exposure.

### miR342 involvement in biological processes related to survival

To gain more insight into the biological significance of miR342 we conducted several bioinformatic analyses. First, we looked at its target genes using ClueGO Cytoscape plugin software. As shown in Table 2, some of its target categories include regulation of viable cell number, cell proliferation and cell death.

**Table 2:** Biological processes implicated for miR342.

ID	Term	P-value
GO:0008284	Positive regulation of cell proliferation	< 0.0001
GO:0048146	Positive regulation of fibroblasts proliferation	< 0.0001
GO:0048144	Fibroblast proliferation	1E−04
GO:0048145	regulation of fibroblasts proliferation	1E−04
GO:0048522	Positive regulation of cellular process	1E−04
GO:0045667	Regulation of osteoblast differentiation	1E−04
GO:0042063	Gliogenesis	2E−04
GO:0048518	Positive regulation of cellular process	2E−04
GO:0001649	Osteoblast differentiation	3E−04
GO:0001649	Cell proliferation	4E−04

To further understand potential miR-342 targets involved in cell proliferation and cell cycle, we used several bioinformatic tools which identify putative miR binding sites based on the homology between the 3'UTR sequence of genes and miR sequences. The analysis was done by using TargetScan^20^, miRBase^21^, DIANA miRPath^27^, miRDB^24^ and miRWalk^25^. From the results of this analysis we selected six genes that may be biologically relevant to cell proliferation. These include: RAS1, NKAP and RGS4. In addition, genes that are involved in epigenetic regulation may also serve as targets for miR342. These include the following: KMT2A-a lysine (K)-specific methyltransferase 2A, HAT1-histone acetyltransferase 1, and the TRMT2A-a tRNA methyltransferase 2 homolog A. These proteins are all histone methylation modifying enzymes which mediate gene expression, genomic stability and mitosis^36,37^.

**Figure 8:**
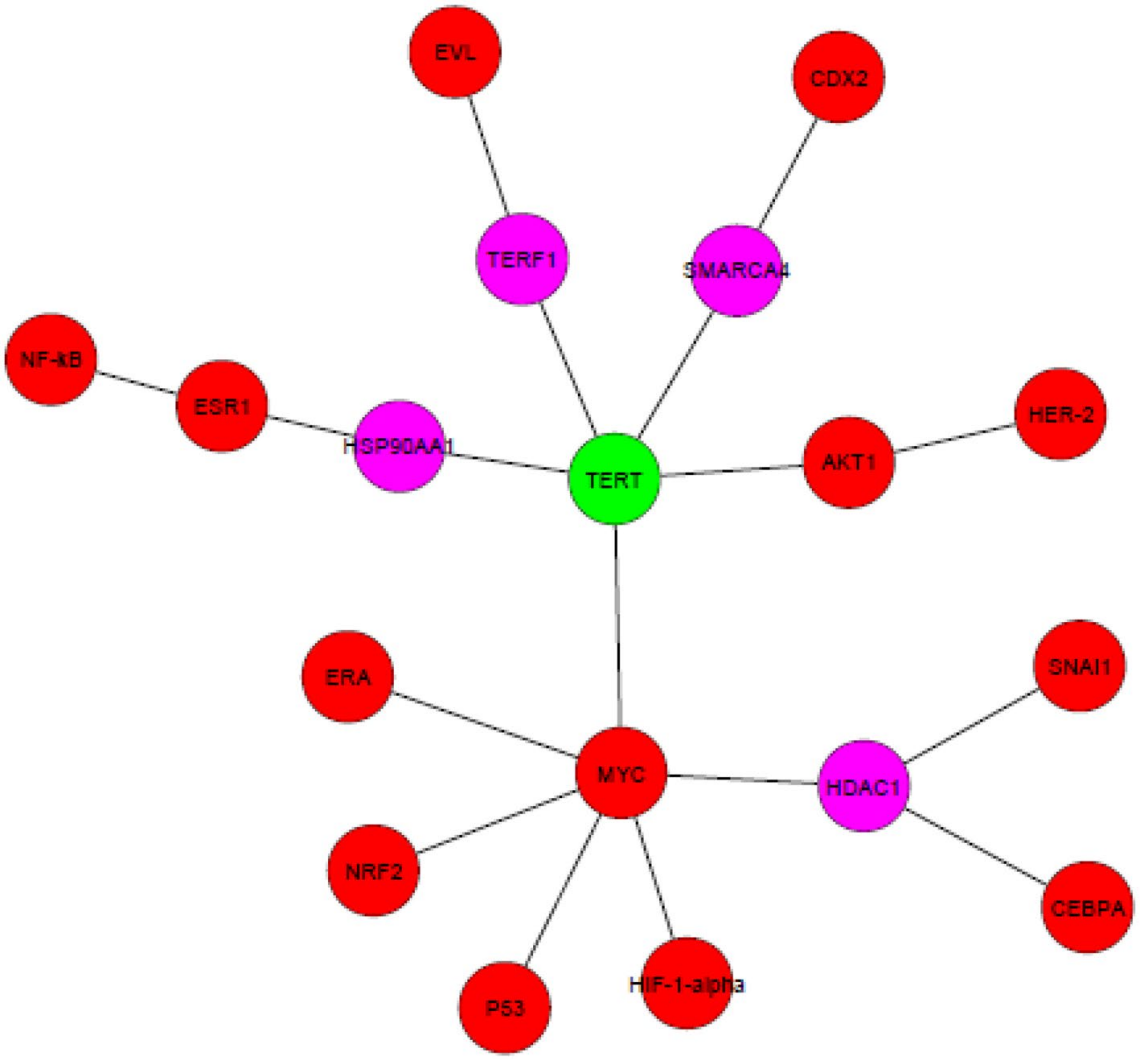
ANAT output of four miRNA target proteins and telomerase. Pink circles represent proteins identified by ANAT. Red circles are the targets of the miRs predicted by miRDB. The green circle refers to the anchor telomerase (hTERT).

We have used the ANAT software here as well, to understand possible associations between the four selected miRs and telomerase. Since ANAT is a protein- protein based network, we first identified the protein regulators of each miR and then applied ANAT using telomerase as the anchor and the regulators as end nodes. The regulators are depicted in Table 3. Figure 8 depicts the network formed by ANAT. These results point to different connectivity of telomerase and the protein regulators of the four miRs, reflecting a possible differential biological importance of each miR in our setting.

**Table 3:** Protein regulators of the four selected miRs.

miR symbol	upstream regulators	References
hsa-miR-92a-3p	E2F, C Myc-↑	Oncotarget; 2020, 39: 6529–43
hsa-miR-128-3p	HIF-1α- ↑	Aging (Albany NY) 2020, 12: 4067–81 Cancer Res. 2012, 72: 6036–50
	Snail- ↑	Oncotarget 2017, 8: 39,280–39,295
	P53R 175H- ↑	Carcinogenesis, 2010, 31: 1045–1053
hsa-miR-342-3p	HER2Δ16- ↑	Mol Cancer 2010, 9:317
	EVL (Ena/VASP-like)- ↑	Scientific Reports 2018, 8:12,252
	Estrogen Receptor (ER)- ↑	Asian Pac J Cancer Prev 2012, 13: 873–7
hsa-miR-125b-1-3p	by CDX2, ↑	Summarized in: Leukemia; 2012, 26: 2011–18
	NF-κβ, P53 ↑ or↓	PLoS One 2011, 6: e17169
		Cell Cycle 2007, 6: 1586–93
	by MYC and AKT1 ↓	Nat Genet 2008, 40: 43–50
	C/EBPa- ↑	Immunity 2009, 31: 220–31
	Era- ↑	Leukemia 2015, 29: 2402 – 2448
	Nrf2- ↑	J. Hepatol. 2015, 63: 1466–75
		Cell Death Differ 2015, 4: 654–64

**↑** and **↓** indicate the directionality of the regulation of miR125b-1 by the various regulators.

Finally we performed Gene Ontology (GO) terms analysis to shed more light on the putative functions of the four upregulated miRs in this setting (Supplemental Table 1). miRNAs were analyzed with DAVID functional Annotation tool to identify GO categories that are mostly represented in this subset. The results of this analysis demonstrated that the targets of the four miRs modulate the expression of genes that are implicated among other functions to mitotic cell cycle. Other gene products are related to protein, DNA and RNA metabolism.

Taken together, these analyses highlight both the putative biological connections between the miRs that were upregulated by exosomal telomerase and telomerase itself and the relevance of at least some of these miRs to proliferation processes.

## Discussion

The importance of the cancer microenvironment in cancer progression and aggressiveness is well established. Tumor cells manipulate their microenvironment to support cancer by modulating stromal cells and the extra cellular matrix^38,39^. The intercellular communication between cancer cells and stromal cells, especially the one mediated by cancer-associated fibroblasts (CAFs) can be carried out, among others, by exosomes^40^. Telomerase activity has been detected in approximately 90% of tumor samples of all types and is a central regulator of the hallmarks of cancer^41^. The importance of telomerase activity in cancer biology motivated us to study its putative role in the crucial interactions between cancer cells and their surrounding microenvironment. In a previous study we showed that the transcript of telomerase is packaged in exosomes derived from cancer cell. This transcript is subsequently taken up by recipient cells, where it is translated and assembled into a fully active enzyme and its activity provides the cells with a survival advantage. In the current study we report two major biological processes that are regulated by exosomal telomerase in exposed fibroblasts: the upregulation of CAF associated markers and modulation of the microRNA landscape of these cells.

### The induction of CAF markers

Numerous recent studies reported the transformation of fibroblasts to CAFs after exposure to cancer cells derived exosomes^42–44^. We examined the expression of two widely used CAFs markers in exosome treated fibroblasts: αSMA and Vimentin. αSMA is the most widely used CAFs and myofibroblasts marker^45^. It is one of six different actin isoforms and was found to increase fibroblasts’ contractile ability. We found a telomerase activity dependent trend of increase in αSMA levels in treated fibroblasts 48 h post Jurkat cells derived exosomal exposure. The lack of statistical significance may be attributed to the fact that the levels of that marker were assessed in the total fibroblast population and not in the sub-fraction of the cells that underwent CAFs transformation, or alternatively to the number of exosomes used in our setting which could potentially be scaled up. Another explanation may be attributed to the dependency of αSMA levels on the expression levels of TGFβ in the cancer cells derived exosomes to which the fibroblasts were exposed as recently reported^35^. It may well be that our purified exosomes possessed a low level of TGF $\upbeta$.

Vimentin, a Type III intermediate filament, is expressed in mesenchymal stem cells, the origin of fibroblasts and confers mechanical stability and contributes to cell motility^46,47^. Exposure of the fibroblasts to Jurkat cells derived exosomes elevated the levels of vimentin during 24 h of exosomal exposure. This effect lasted 48 h and may be attributed at least in part to telomerase activity since cells with telomerase inhibition exhibited a milder effect, although non- statistically significant. The lack of the statistical significant may have been overcome by upscaling exosomal concentration (in this regard please see later the limitation of the in vitro setting). We also observed additional bands of vimentin after 48 h of exosomal exposure that can arise from different isoforms of vimentin or posttranslational modifications^48,49^. The connection found by ANAT strengthens the experimental results and point to another extracurricular activity of telomerase.

IL-6 is a multifunctional cytokine that plays a central role in the regulation of inflammatory processes and is a prototype of an angiogenic related cytokine^50,51^. Many studies reported the secretion of IL-6 from CAFs^52,53^ and its various tumor promoting downstream effects. In our setting, we found a clear trend of increase in IL-6 secretion levels from the exosomally exposed fibroblasts. Again, the effect was telomerase mediated to some degree since it was milder after inhibiting telomerase in the recipient fibroblasts by GRN163L. This effect can be mediated by TERT in a non-canonical pathway as IL-6 has been previously shown to be a direct target of telomerase activity^54^.

Having said that, we must recall that the exosomal- based in vitro study employing exosomes is artificial in two ways: firstly, the concentrations of exosomes travel in the blood may be totally different from that used in vitro. Second, we apply exosomes in vitro only once but the in vivo flow of exosomes is continuous. In the light of these differences the question of kinetics of telomerase activity, IL-6 and the CAF markers should be addressed in future studies. The other yet unanswered question is related to the existence of telomerase activity in CAFs in vivo. This point should be further addressed in future studies as well. Even if CAFs do not possess telomerase activity, it is possible that the activity of the enzyme occurred as a transient stage during the actual formation of cancer associated fibroblasts from intact ones. Interestingly, one report has found similar telomere dynamics of HCC and CAF cells, while in HCC cells telomerase activity was found^55^. Whether and how this may indirectly reflect somewhat similar telomerase activation in the HCC associated CAF cells is to be studied as well.

### Exosomal hTERT affects the levels of microRNA in recipient fibroblasts

The other part of our study describes the changes in miR expression in response to exosomal telomerase in these fibroblasts.

miRNAs have been the focus of many biological studies in general and in the field of cancer research in particular as regulators of a large number of target genes, both as tumor suppressor or drivers. Given the fact that a single miRNA can target a large number of mRNA transcripts, aberrant expression of a set of miRNAs could have a significant effect on the cellular function by affecting multiple signaling pathways in the cells. Moreover, miRNAs are key regulators of the tumor-promoting functions and it is becoming clearer that cancer-directed changes regulate these miRNA based networks‏.

The rational for this part of our study was based on a recent report showing that telomerase activity was implicated in regulating the biogenesis of microRNAs^16^. To study the putative effect of exosomal telomerase on the expression of microRNAs in the recipient fibroblasts we exposed fibroblasts to Jurkat cells derived exosomes under the inhibition of telomerase activity in the recipient cells and analyzed the resulting microRNA landscape. We compared the levels of these micrRNAs to those of control intact fibroblasts, Jurkat cells and fibroblasts expressing hTERT. Using that analysis we identified four miRs that were significantly overexpressed in fibroblasts exposed to exosomes containing hTERT compared to intact fibroblasts: miR-342-3p, miR-125b-1, miR-128-3p and miR-92a. By Q-PCR we validated the results of our analysis. These microRNAs were not expressed when telomerase activity was inhibited in the recipient cells and are involved in cancer related processes^56^.

We also observed that the inhibition of hTERT was associated with dysregulation of miRNAs, which in turn could influence translation and function of over 600 gene targets‏.

By comparing the expression of microRNAs in fibroblasts which stably expressed hTERT to the intact fibroblasts we identified a different set of microRNAs, with miR-146a showing the highest expression level in the presence of hTERT (20 fold). miR146 is implicated as a pleiotropic regulator of carcinogenesis^57^. The difference between the results of the two comparisons: that of exosomal hTERT versus that of ectopically expressed hTERT may stem from differences in the two experimental systems, for example, the expression levels of the gene.

### Active packaging of miRs into exosomes

Interestingly, microRNA expression profile analysis of exosomal microRNAs derived from Jurkat cells identified some miRNAs that were expressed at higher levels in exosomes than in their parent cells, implying on an active packaging of molecules into exosomes and thus might have an important biological role in some setting. However, the mechanisms for selective packaging and release of exosomal microRNAs are currently unknown and may be affected by the malignant transformation process itself. As an example we identified miR-451a which is implicated in regulation of tumor growth^58^, which was highly expressed in Jurkat cells derived exosomes versus the parental donor cells.

### miR342

In light of its higher expression and its biological role in carcinogenesis, miR-342 was chosen for the following parts of our study. To understand whether this miR is involved in the phenotypic changes induced by exosomal telomerase^14^ the chosen miR was overexpressed in fibroblasts and we studied the putative effect on cell proliferation and cell cycle status. miR342 ectopic expression induced a 50% increase in cell proliferation which is reflected by an increase of about two-fold in the S phase of the cell cycle of these cells suggesting that miR342 may act in the transition from G1 to S phase‏. These results are in accordance with the above-mentioned cancer related functions of that miR^59,60^. Of note, this miR was also expressed in Jurkat cells but when analyzing the RNA cargo of their secreted exosomes we detected only a small, non-statistically significant amount of this miR. Since recipient cells in which telomerase was inhibited did not express miR342, we concluded that its activity is needed to activate the expression of miR342. Since this expression was detected 24 h post exosomal exposure, a too short time to enable telomere elongation, we reasoned that this is another example of a non- canonical type of telomerase activity (This type of the enzyme's activity is described hereafter). Of note, the extracurricular activity of telomerase is dependent either on hTERT or its RNA subunit, hTERC. Since exosomal hTERT was introduced to the recipient fibroblasts which then complexes with the cellular hTERC, we were unable to discriminate whether the induction of miRs was dependent on hTERT or the activity of telomerase in our setting.

The results of our study may explain, at least in part, the survival effects provided by exosomal telomerase to the fibroblast recipient cells. Interestingly, the GO functions of the predicted target genes of the four miRs that were upregulated by exosomal telomerase, Toll Like Receptors (TLR) pathways, are in line with the increase in proliferation obtained in our setting (supplemental Table 1). The activation of TLR is implicated in the development and perpetuation of tumor cells by various mechanisms including the secretion of various cytokines promoting proliferation. The findings of CAF associated markers in response to exosomal telomerase at the first part of the study, especially the secretion of IL-6, fits well with the putative activation of TLR in inducing this cytokines secretion to promote tumor growth.

When analyzing miR342 gene targets an enrichment of gene pathways related to proliferation was obtained. These include NKAP, RGS4 and RASA1 pathways. RASA1 acts as a tumor suppressor^61^; thereby its downregulation may promote cell proliferation through the RAS-RAF-MEK-ERK pathway^62–64^. Similarly, the downregulation of NKAP may lead to cellular proliferation via the NF-kappa-B signaling pathway. Other predicted target genes of miR342 were implicated in epigenetic regulation. These include HAT1-histone acetyltransferase 1 and KMT2A-a lysine (K)-specific methyltransferase 2A, both involved in histone methylation implicated in malignant transformation^65^. Other pathways related to the targets of miR342 include the TGF-beta, the NF-κB and the p38/MAPK and cytokine-cytokine receptor interaction pathways, all highly related to the proliferative effect obtained by miR342.

### Telomerase non-canonical pathways

In addition to telomere maintenance, telomerase also performs other biological roles which are defined as "extracurricular". Acting via NFκB and Wnt/β-catenin pathways, these activities involve, among others, prevention of apoptosis, growth parameters and miRs biogenesis^66^. Here we show another side of the extracurricular activities of the enzyme, namely- the activation of miRs in exosome recipient cells. The interpretation of this result is supported by the analysis of ANAT, showing multiple connections between the four miR targets that were upregulated by the shuttle of exosomal telomerase and the enzyme itself.

The main limitation of the current study is its in vitro artificial setting. In order to clarify the implications of our results, further study should measure exosome concentrations in cancer patients' sera and compare it to the exosome concentrations used in our study. Another difference between the in vitro and in vivo setting is the continuous nature of the process of secretion and uptake of exosomes in the human body versus a single dose exposure in vitro. Therefore, an in vivo study based on an animal model is needed. A major methodology limitation in the current study is the luck of using somatic cells derived exosomes as a control group. Although somatic cells do not possess telomerase activity it should be done in a future work. Another pitfall of this study is the luck of specific follow up of the effect of GRN163L, the specific inhibitor of telomerase which acts as an inhibitor of the hTR, (known also as Imetelstat). However, in our laboratory we have examined the effect of GRN163L on telomerase activity in numerous other studies and we have obtained almost 90% inhibition of the activity by this specific inhibitor).

Clarifying these exosomal telomerase dependent microenvironmental relationships may be exploited in the future for the development of new rationalized drugs against many types of cancer.

In summary, our study shows that hTERT exosomes alter the phenotype and the microRNA transcription profile of the recipient fibroblasts. Specifically, two CAF related markers were upregulated, one studied cytokine was secreted and four microRNAs including miR342. These changes may promote the exposed fibroblasts to support cancer progression through exosomal telomerase.

## Supplementary Information

Supplementary Information 1.

Supplementary Information 2.

Supplementary Information 3.
