# Supplementary material for: Exosomal telomerase transcripts reprogram the microRNA transcriptome profile of fibroblasts and partially contribute to CAF formation
Source: Sci Rep. 2022 Sep 30;12:16415. doi: 10.1038/s41598-022-20186-8 (PMC9525320; doi:10.1038/s41598-022-20186-8)
Supplement: Supplementary file 1 — Supplementary Information 1. [file 41598_2022_20186_MOESM1_ESM.docx]

Table 1.

| Go Category | | *P Value* | Number of genes |
| --- | --- | --- | --- |
| ***mitotic cell cycle*** | | *<1e-325* | *97* |
| *Fc-epsilon receptor signaling pathway* | 5.55E-16 | | 46 |
| *DNA metabolic process* | 1.67E-15 | | 143 |
| *protein complex assembly* | 6.33E-15 | | 120 |
| *epidermal growth factor receptor signaling pathway* | 9.44E-15 | | 59 |
| ***fibroblast growth factor receptor signaling pathway*** | 3.94E-10 | | 50 |
| *cytoskeletal protein binding* | 6.32E-10 | | 108 |
| *transcription, DNA-templated* | 7.00E-10 | | 299 |
| *enzyme regulator activity* | 1.36E-09 | | 133 |
| *cellular lipid metabolic process* | 4.17E-09 | | 33 |
| *RNA splicing* | 1.58E-08 | | 60 |
| *translational termination* | 5.53E-08 | | 26 |
| *chromatin organization* | 6.71E-08 | | 31 |
| *SRP-dependent cotranslational protein targeting to membrane* | 6.74E-08 | | 30 |
| ***G2/M transition of mitotic cell cycle*** | 8.37E-08 | | 34 |
| *nuclear-transcribed mRNA catabolic process, nonsense-mediated decay* | 1.50E-07 | | 31 |
| *protein targeting* | 2.66E-07 | | 64 |
| *transcription factor binding* | 8.87E-07 | | 93 |
| ***G1/S transition of mitotic cell cycle*** | 2.13E-06 | | 39 |
| *transcription initiation from RNA polymerase II promoter* | 2.50E-06 | | 37 |
| *Fc-gamma receptor signaling pathway involved in phagocytosis* | 3.15E-06 | | 16 |
| *ribonucleoprotein complex assembly* | 4.96E-06 | | 31 |
| *immune system process* | 1.47E-05 | | 168 |
| *mRNA processing* | 2.28E-05 | | 74 |
| *translational elongation* | 2.65E-05 | | 30 |
| ***cell cycle*** | 4.77E-05 | | 127 |
| *phosphatidylinositol-mediated signaling* | 6.75E-05 | | 20 |
| *mRNA splicing, via spliceosome* | 9.46E-05 | | 40 |
| *post-Golgi vesicle-mediated transport* | 0.000137 | | 13 |
| *nucleocytoplasmic transport* | 0.000162 | | 55 |
| ***mitotic nuclear envelope disassembly*** | 0.000241 | | 11 |
| *negative regulation of transcription from RNA polymerase II promoter* | 0.000283 | | 137 |
| *positive regulation of ubiquitin-protein ligase activity involved in mitotic cell cycle* | 0.00029 | | 17 |
| *TRIF-dependent toll-like receptor signaling pathway* | 0.000339 | | 15 |
| ***regulation of ubiquitin-protein ligase activity involved in mitotic cell cycle*** | 0.000435 | | 17 |
| *toll-like receptor 10 signaling pathway* | 0.000441 | | 14 |
| *transcription from RNA polymerase II promoter* | 0.000652 | | 79 |
| *cellular component movement* | 0.00067 | | 22 |
| *translational initiation* | 0.000709 | | 40 |
| *toll-like receptor TLR1:TLR2 signaling pathway* | 0.000978 | | 14 |
| *toll-like receptor TLR6:TLR2 signaling pathway* | 0.000978 | | 14 |
| *transforming growth factor beta receptor signaling pathway* | 0.000989 | | 31 |
| *regulation of cellular amino acid metabolic process* | 0.001093 | | 13 |
| *protein binding, bridging* | 0.00126 | | 19 |
| *generation of precursor metabolites and energy* | 0.001336 | | 46 |
| *transcription corepressor activity* | 0.001417 | | 42 |
| ***anaphase-promoting complex-dependent proteasomal ubiquitin-dependent protein catabolic process*** | 0.001489 | | 19 |
| *toll-like receptor 5 signaling pathway* | 0.001958 | | 14 |
| *toll-like receptor signaling pathway* | 0.002671 | | 20 |
| *tRNA metabolic process* | 0.003789 | | 29 |
| ***microtubule organizing center*** | 0.003933 | | 60 |
| *stress-activated MAPK cascade* | 0.004027 | | 13 |
| *focal adhesion* | 0.004172 | | 80 |
| *toll-like receptor 3 signaling pathway* | 0.005783 | | 15 |
| *ubiquitin protein ligase binding* | 0.006687 | | 49 |
| *protein polyubiquitination* | 0.008025 | | 30 |
| *'de novo' posttranslational protein folding* | 0.008137 | | 11 |
| *toll-like receptor 4 signaling pathway* | 0.008363 | | 17 |
| *cofactor metabolic process* | 0.009382 | | 35 |
| *toll-like receptor 9 signaling pathway* | 0.01151 | | 13 |
| *ligase activity* | 0.017493 | | 79 |
| *COPI coating of Golgi vesicle* | 0.017988 | | 6 |
| *phosphatidylinositol biosynthetic process* | 0.018825 | | 13 |
| *toll-like receptor 2 signaling pathway* | 0.020821 | | 14 |
| *DNA methylation* | 0.037828 | | 8 |

**Supplamented Table 1.** The Most significant GO categories that were correlated with miR-342-3p, miR-125b-1, miR-128-3p and miR-92a-3p expression **(PV < 0.001).**
