# Supplementary material for: Exosomal telomerase transcripts reprogram the microRNA transcriptome profile of fibroblasts and partially contribute to CAF formation
Source: Sci Rep. 2022 Sep 30;12:16415. doi: 10.1038/s41598-022-20186-8 (PMC9525320; doi:10.1038/s41598-022-20186-8)
Supplement: Supplementary file 2 — Supplementary Information 2. [file 41598_2022_20186_MOESM2_ESM.docx]

**Supplementary Figure legend**

Supp. Fig. 1. Original blots from which Fig. 4 was prepared by cropping.

A. The blot used for the αSMA (lanes 2-7). Please note that the molecular weight of the reference gene tubulin is higher than that of the tested protein αSMA and therefore the blot was cropped and re arranged. B. The blot used for tubulin (first 6 lanes). All calculations of the relative levels of αSMA using tubulin as references were conducted on blots from the same experiments. C. The original blot used to form Fig 4B.

Supp. Fig. 2: Analysis of relative microRNA expression after Jurkat derived exosomal exposure. A. Identification of miRNAs which were differentially expressed in pHFF cells incubated with exosomes from Jurkat cells (y-axis) versus pHFF cells with no exosomal exposure (x-axis). Each blue cross represents a miRNA; Grey crosses are probes not tested in the analyses (e.g. control sequences or low expression). Each cross signifies the median expression of a probe in the two groups; Red circles: miRs with fold-change > 2, p<0.05

B. Correlation plot between miRNA expression in pHFFs incubated with exosomes + telomerase inhibitor GRN163 (y-axis) vs. pHFF incubated with exosomes only (x-axis). Upregulated miRs were enriched for G-rich miRs (technical bias), so additional filters were used for this comparison. In black circled yellow: fold-change > 2, p-value < 0.05 and miRs that are not G-rich.

C. Correlation plot between miRNA expression in pHFFs incubated with exosomes + telomerase inhibitor GRN163 (y-axis) vs. pHFF alone (x-axis). Diagonal lines demarcate 1.5-fold difference levels in expression between the two groups; In blacked circled yellow: p<0.05 with the largest fold-change (fold-change > 5). Grey crosses are probes not tested in analyses (e.g. control sequences or low expression).

D. Correlation plot between miRNA expression in pHFFs incubated with exosomes + telomerase inhibitor GRN163 (y-axis) vs. pHFF exposed to exosomes only (x-axis).

E. miRNA expression in pHFF cells transfected with the hTERT gene (y-axis) vs pHFF wild type cells (x-axis). Bold red circles depict a differential change of >2.
