# Supplementary material for: Exosomal telomerase transcripts reprogram the microRNA transcriptome profile of fibroblasts and partially contribute to CAF formation
Source: Sci Rep. 2022 Sep 30;12:16415. doi: 10.1038/s41598-022-20186-8 (PMC9525320; doi:10.1038/s41598-022-20186-8)
Supplement: Supplementary file 3 — Supplementary Information 3. [file 41598_2022_20186_MOESM3_ESM.pptx]

## Slide 1
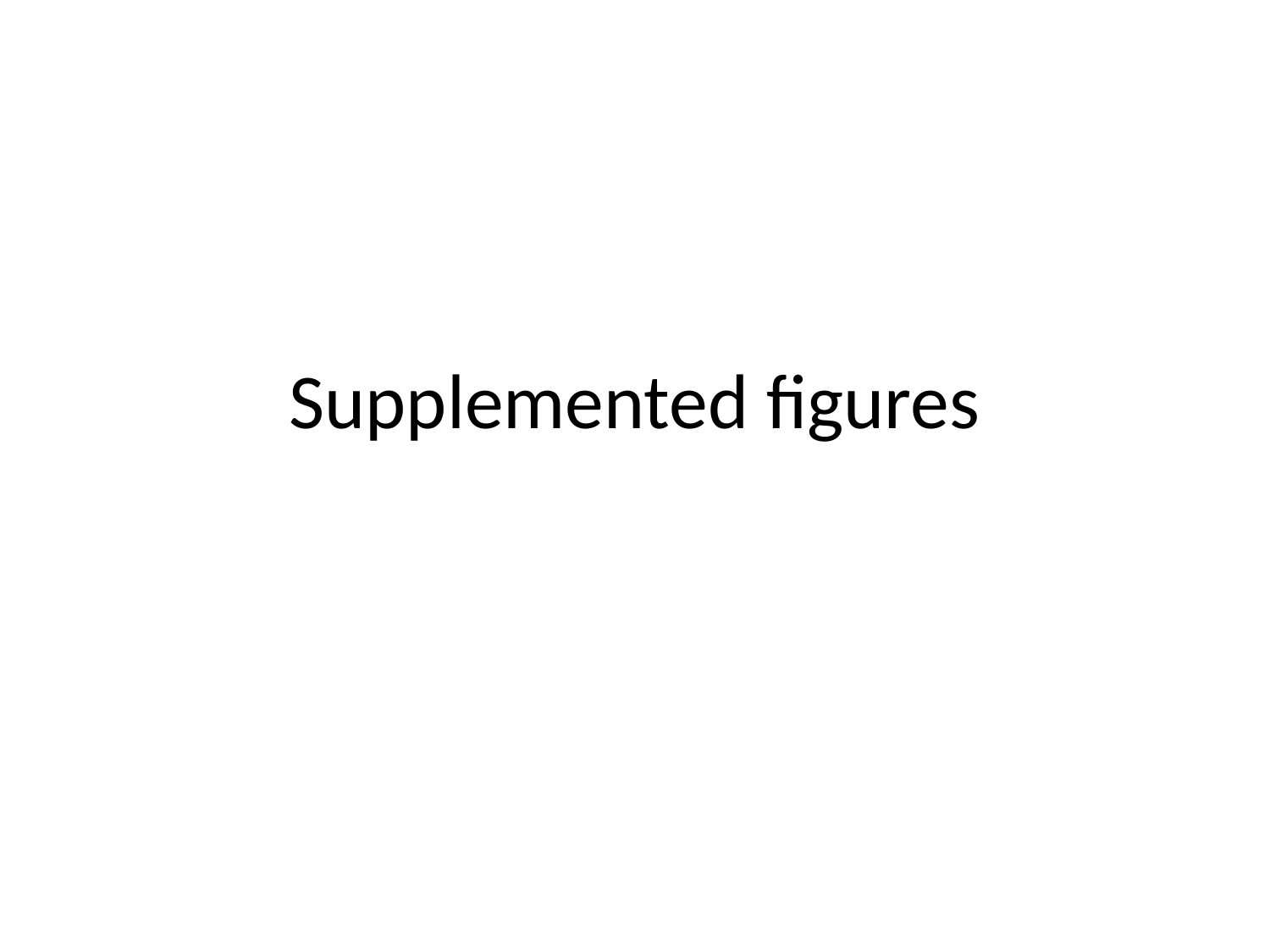

# Supplemented figures

## Slide 2
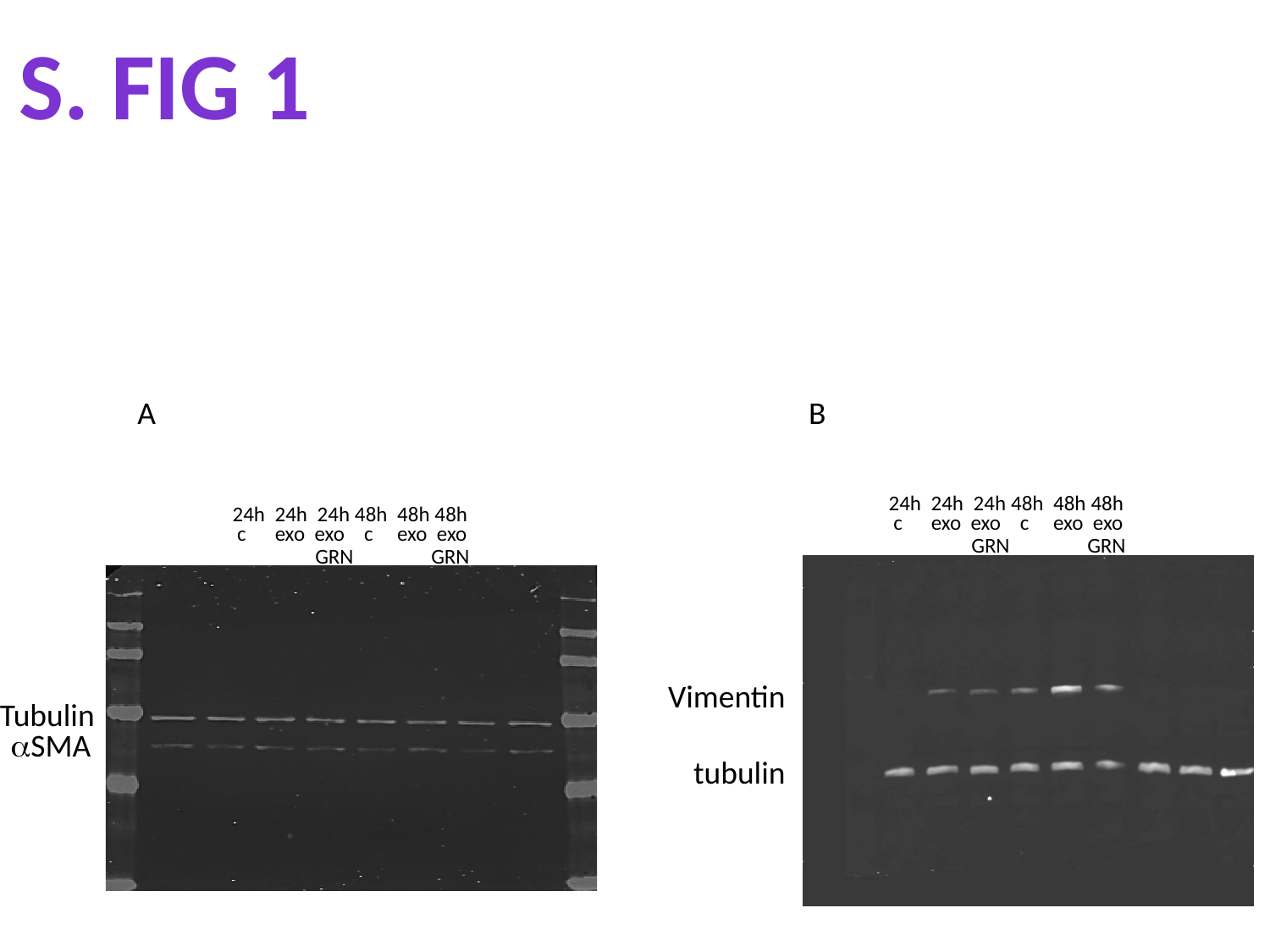

S. Fig 1
A B
24h 24h 24h 48h 48h 48h
 c exo exo c exo exo
 GRN GRN
24h 24h 24h 48h 48h 48h
 c exo exo c exo exo
 GRN GRN
Vimentin
tubulin
Tubulin
aSMA

## Slide 3
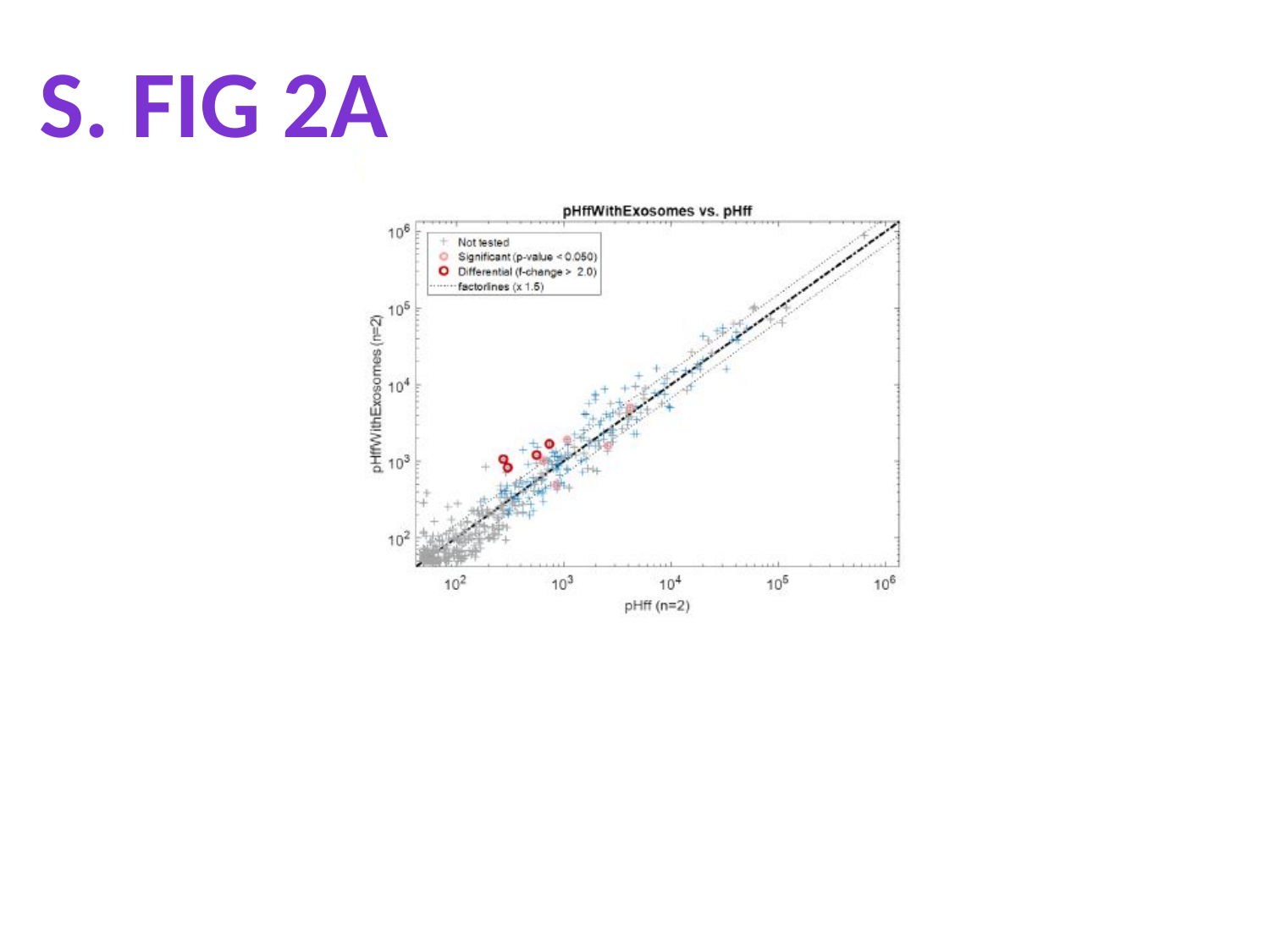

S. Fig 2A

## Slide 4
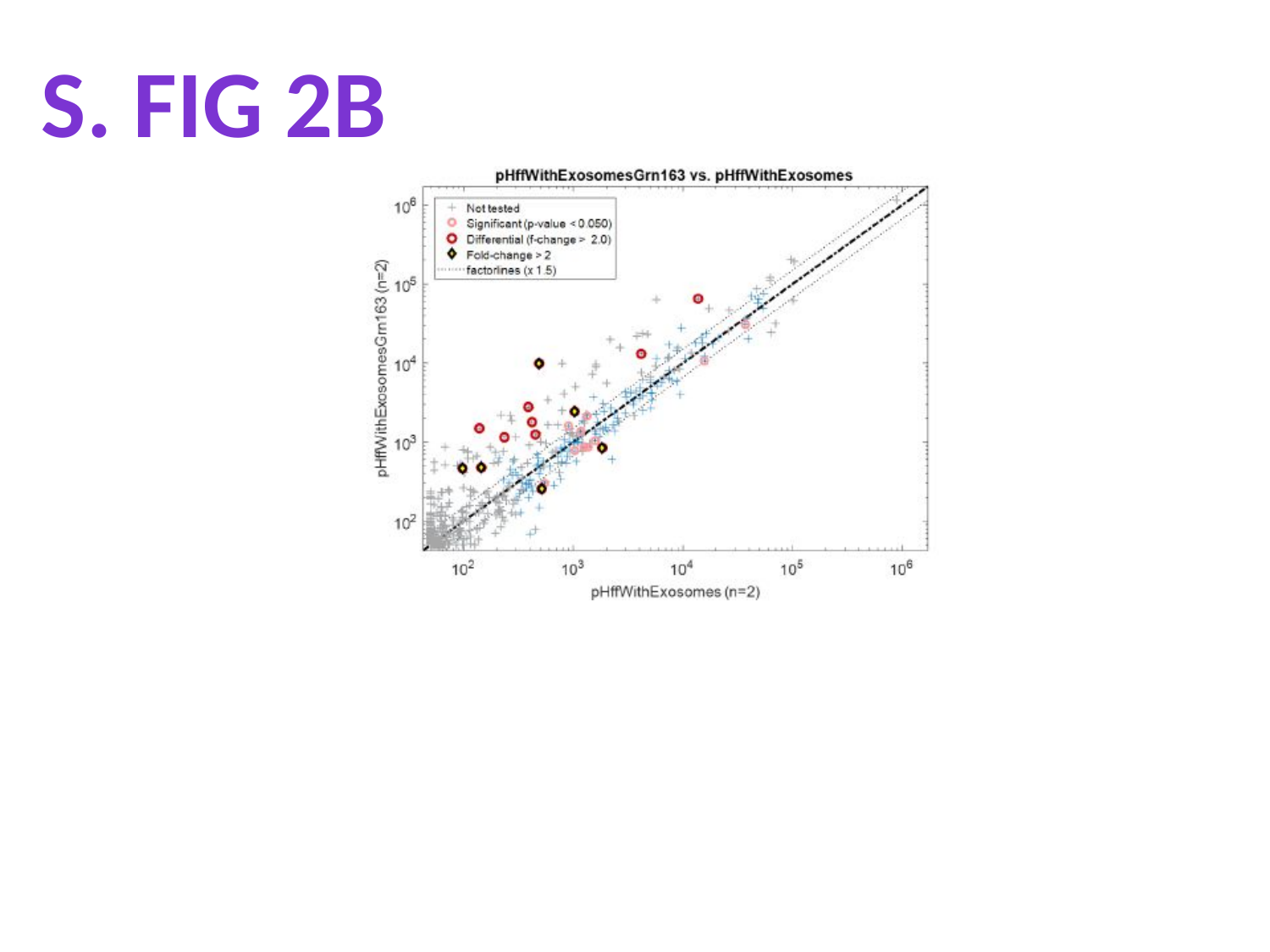

S. Fig 2B

## Slide 5
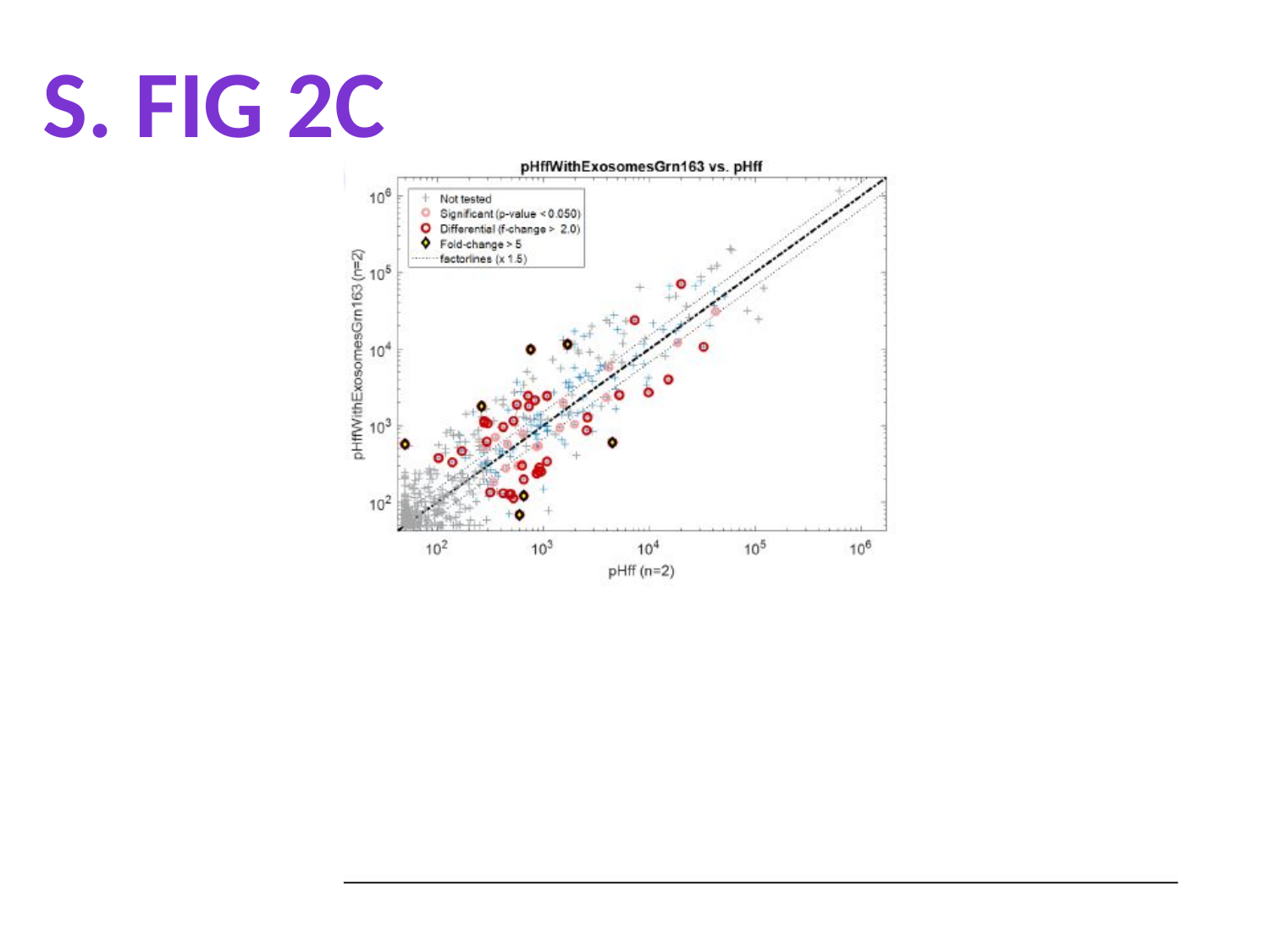

S. Fig 2C

## Slide 6
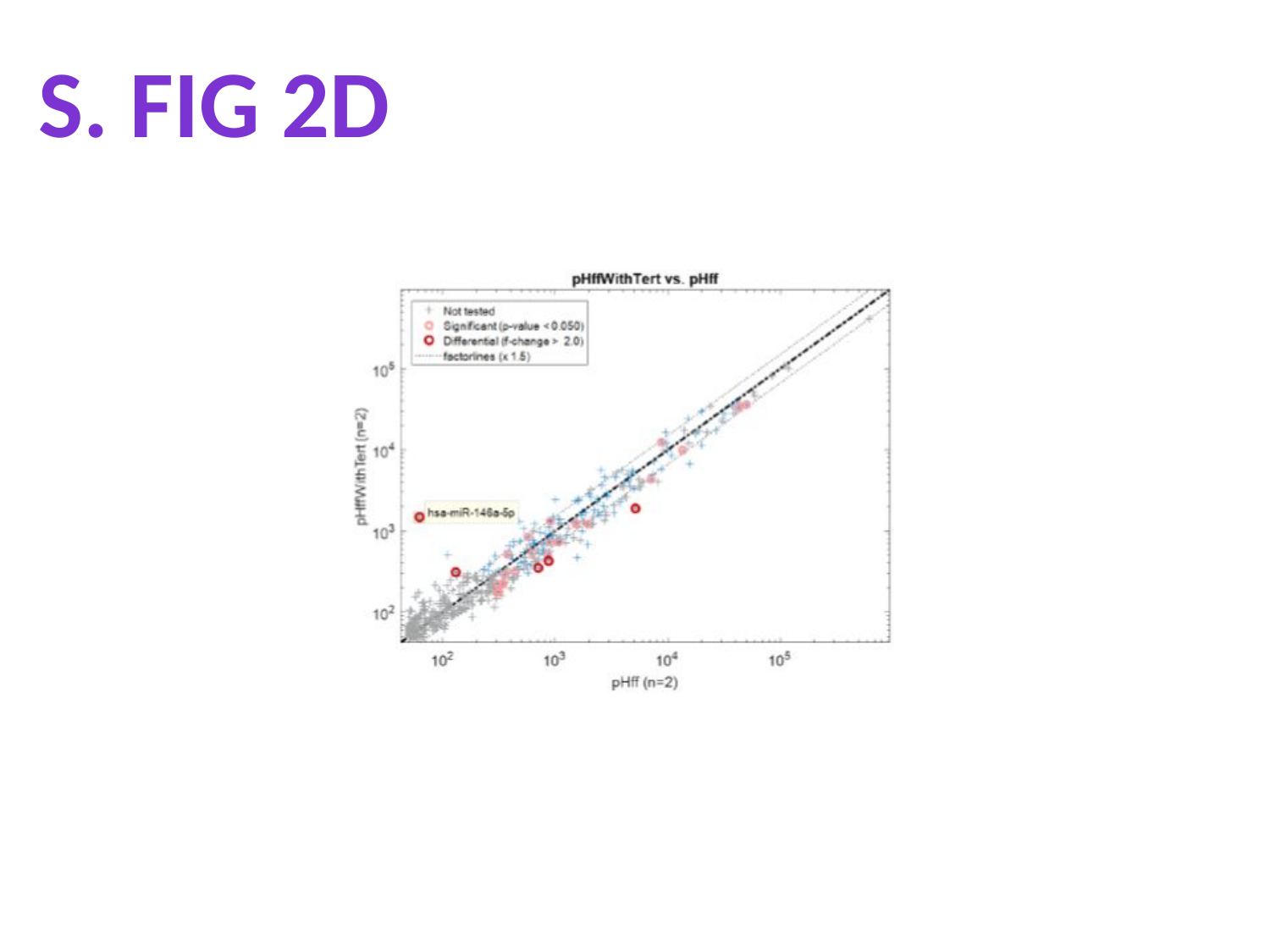

S. Fig 2D

## Slide 7
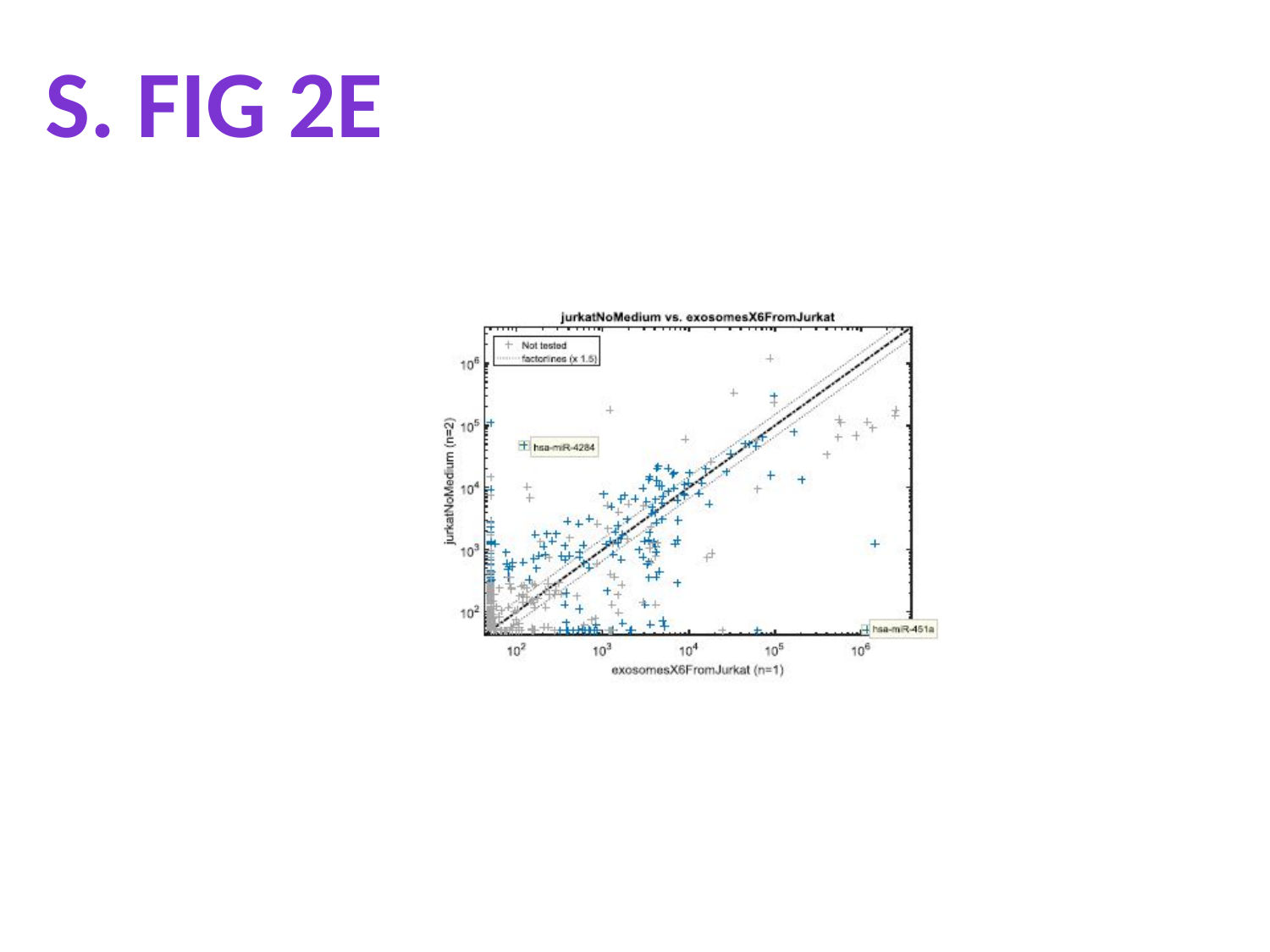

S. Fig 2E
